# Application of LC-MS/MS methods and risk assessment for selected emerging contaminants and their transformation products in a sub-Adriatic river system

**DOI:** 10.1007/s10661-026-15437-4

**Published:** 2026-06-04

**Authors:** Aleksander Kravos, Helena Prosen

**Affiliations:** University of Ljubljana, Faculty of Chemistry and Chemical Technology, Večna pot 113, 1000 Ljubljana, Slovenia

**Keywords:** Chemical fingerprinting, Emerging pollutants, Solid-phase extraction, Spatial trends, Surface water

## Abstract

The present study investigates solid-phase extraction (SPE) followed by LC-MS/MS for the analysis of contaminants of emerging concern (CECs) and transformation products (TPs) in the Slovenian-Italian Vipava river system (part of the Adriatic Sea catchment). The adapted method enabled quantitative analysis of 13 various CECs and qualitative analysis of 2 UV filters. SPE recoveries ranged from 17 to 116%, LODs from 0.11 to 5.9 ng L^−1^, and overall %RSDs were below 20%. A separate LC-MS/MS method enabled analysis of 42 suspect TPs. Total-ion current LC-MS chromatograms (chemical fingerprints) for river water extracts were also recorded to observe changes along the river course. Ten CECs were frequently detected, and two rosuvastatin TPs, ROS349 and ROS481, were both identified by LC-MS/MS and confirmed with LC-QTOF. Caffeine was the most frequently detected analyte, with average concentrations around 100 ng L^−1^, and was even detected in spring water from karst aquifers. Most other CECs were present at higher concentrations near wastewater treatment plants (WWTPs) and landfills. The influence of these two facilities was also observed through chemical fingerprinting. Acesulfame and tris(2-ethylhexyl) phosphate occasionally exceeded 100 ng L^−1^. The latter, along with diflufenican (maximum 67 ng L^−1^), resulted in elevated environmental risk quotients. Avobenzone and octocrylene showed higher abundance near WWTPs. Telmisartan and rosuvastatin were quantified at concentrations below 73 and 18 ng L^−1^, respectively. Overall, this study expands knowledge of anthropogenic stress on the surface waters in the part of the sub-Adriatic region.

**Supplementary Information:**

The online version contains supplementary material available at 10.1007/s10661-026-15437-4.

## Introduction

Advances in liquid chromatography-tandem mass spectrometry (LC-MS/MS) have enabled ultra-trace analysis and the discovery of contaminants of emerging concern (CECs) in natural waters. Their widespread presence in freshwaters raises concerns due to limited knowledge of their environmental fate, behaviour, and (eco)toxicity. The same analytical procedures that enabled the discovery of CECs are now used to study, understand, prioritise, and control them (Gavrilescu et al., 2015). Off-line solid-phase extraction (SPE) followed by LC-MS/MS is one of the most preferred analytical procedures providing detection in the ng L^−1^ concentration range and relatively low matrix effects (Klančar et al., 2018). In recent years, transformation products (TPs) have also become a subject of interest. Multi-residue target analyses of CECs are being supplemented with suspect screening of numerous known or predicted TPs with different chemical properties. Therefore, a generic SPE approach is often applied (Maculewicz et al., 2022; Sadutto & Picó, 2020).

Apart from CECs (ultra-trace or nano-contaminants) analysed with LC-MS/MS using multiple reaction monitoring (MRM), river water extracts also contain other background substances, such as humic and fulvic substances, proteins, carbohydrates, bacterial metabolites, and macro-contaminants. Elaboration of total-ion current (TIC) chromatograms, referred to chemical fingerprinting, can provide additional qualitative insights into the presence and types of unknown natural and/or anthropogenic substances in river water, but is poorly represented in the literature. Chemical fingerprinting offers a simple means of detecting changes and alterations in river’s natural chemical fingerprint and aids in identifying contamination sources in a non-specific way. For example, it may serve as a tool to assess the influence of wastewater treatment plants (WWTPs) in the region (Troxell et al., 2024). In addition, comparison between determined concentrations of CECs and pertaining ecotoxicological parameters enables environmental risk assessment (Selak et al., 2022). 

Extensive research on CECs has been conducted over recent decades in European natural waters (García-Córcoles et al., 2019; Gavrilescu et al., 2015); however, noticeably less has been done in Slovenia and in the wider region (Klančar et al., 2018). Slovenia is located at the junction of the Mediterranean and Central European regions. In Slovenia, there is a significant hydrological catchment divide between waters flowing into the Black Sea (80%) in the east and waters flowing into the Adriatic Sea (20%) in the west. The Adriatic Sea catchment in Slovenia covers 3850 km^2^ and is, in physical-geographical terms, considered an exceptionally diverse region with a high proportion of carbonate rocks (Brečko Grubar & Kovačič, 2010).

One of the first studies on CECs in Slovenian surface waters was published by Kosjek et al. (2005), focusing on five target non-steroidal anti-inflammatory drugs. To date, only a few limited studies have focused on the Sava and Drava river systems (Danube river basin) in Central and Eastern Slovenia, respectively. These studies aimed to determine 44 pharmaceuticals (Klančar et al., 2018), multiclass CECs (Česen et al., 2018, 2019; Koroša et al., 2016), 25 endocrine-disrupting compounds (Grobin et al., 2024), and psychoactive drugs of abuse (Verovšek et al., 2023). Data on the occurrence of CECs in Slovenia and its neighbouring countries between 2010 and 2017 were reviewed by Klančar et al. (2018). However, the aforementioned studies primarily address Slovenian river systems within the Black Sea catchment area. There is a significant knowledge gap regarding the occurrence of CECs in western Slovenia, particularly in rivers that flow into the Adriatic Sea, or in the seawater itself. Although priority contaminants are monitored in a few Adriatic rivers within the Slovenian Environment Agency monitoring scheme, data on the occurrence of CECs are scarce for the Slovenian-Italian Soča (Isonzo) and Vipava rivers, as well as for karst groundwaters beneath the surrounding karst plateaus, rivers from the Istra peninsula (Slovenia, Croatia), or rivers in Friuli-Venezia Giulia, Italy (Köck-Schulmeyer et al., 2021; Meffe & de Bustamante, 2014). Only recently, Grobin et al. (2024) analysed natural and synthetic hormones in Slovenian surface waters, including two sampling sites within Vipava river system. In addition, little to no data are available on the occurrence of TPs in Slovenia. This highlights the need for further studies in the region. For instance, the Slovenian-Italian Vipava river system is a good representative of a well-defined sub-Adriatic river, accounting for 16.3% of the total length of surface watercourses in the Slovenian part of the Adriatic Sea catchment (Brečko Grubar & Kovačič, 2010), and was investigated in the present study.

In this work, SPE adaptation for the determination of multiclass CECs and TPs in river water samples is first presented (“Method adaptation and performance” section). The SPE-LC-MS/MS method was applied to study the occurrence of CECs (“Occurrence of CECs” section) and TPs (“Occurrence of transformation products” section) in the Slovenian-Italian Vipava river system. The data were used for the environmental risk assessment to conceptualise the findings and highlight possible environmental concerns (“Environmental risk assessment” section). Finally, chemical fingerprinting of the water samples along the river course enabled tracking of alterations in the river water fingerprint due to the influx of macro-contaminants (“Chemical fingerprinting” section). Overall, this paper addresses the analytical challenges in the analysis of multiclass CECs and their TPs in river water and expands general knowledge on the presence of CECs in defined parts of the European sub-Adriatic region.

## Materials and methods

### Analytes and river samples

Target CECs were selected and previously studied by Kravos and Prosen (2024). The list includes selected representatives from each group of contaminants: high-consumption pharmaceuticals telmisartan (TEL), ramipril (RAM), rosuvastatin (ROS), and simvastatin (SIM); under-researched industrial compounds tris(2-butoxyethyl) phosphate (TBP) and tris(2-ethylhexyl) phosphate (THP); approved UV filters avobenzone (AVO) and octocrylene (OCT); artificial sweeteners cyclamate (CAM), acesulfame (ACS), and neotame (NEO); approved novel pesticides diflufenican (DIF), pirimicarb (PIR), and acetamiprid (ACE); and the anthropogenic tracer caffeine (CAF). Additional compounds were included at the beginning of the study and excluded during its course: moxifloxacin (MOX), 2-mercaptobenzothiazole (MBT), 2-acrylamido-2-methyl-1-propanesulfonic acid (AMA), and cycloxydim (CYC). Further specifications on CECs are provided in Supplementary Materials (SM), Table SM1. A list of suspect TPs was adopted from Kravos et al. (2025), and includes 4 MBT TPs, 6 CYC TPs, 8 ROS TPs, 3 TEL TPs, 10 PIR TPs, 1 DIF TP, 8 MOX TPs, and 2 AVO TPs.

Individual stock solutions of each CEC at 100 mg L^−1^ were prepared in methanol (Honeywell, Chromasolv, LC-MS grade, Muskegon, USA) from commercial standard substances. A multistandard working solution in methanol containing 100 µg L^−1^ of each CEC was then prepared from the stock solutions and used to prepare further ultra-diluted solutions with ultra-pure water (MQ; Millipore, Synergy, Molsheim, France; resistivity = 18.2 MΩ·cm, TOC < 10 ppb). As standards for the identified ROS TPs were commercially unavailable, a pseudo-reference sample was generated. A 5 mg L^−1^ aqueous solution of ROS was photoirradiated with UVA light for 20 min (see the set-up in Kravos et al., 2025). In this way, the pseudo-reference sample contained in situ formed TPs ROS349 and ROS481 necessary for detection confirmation.

**Fig. 1:**
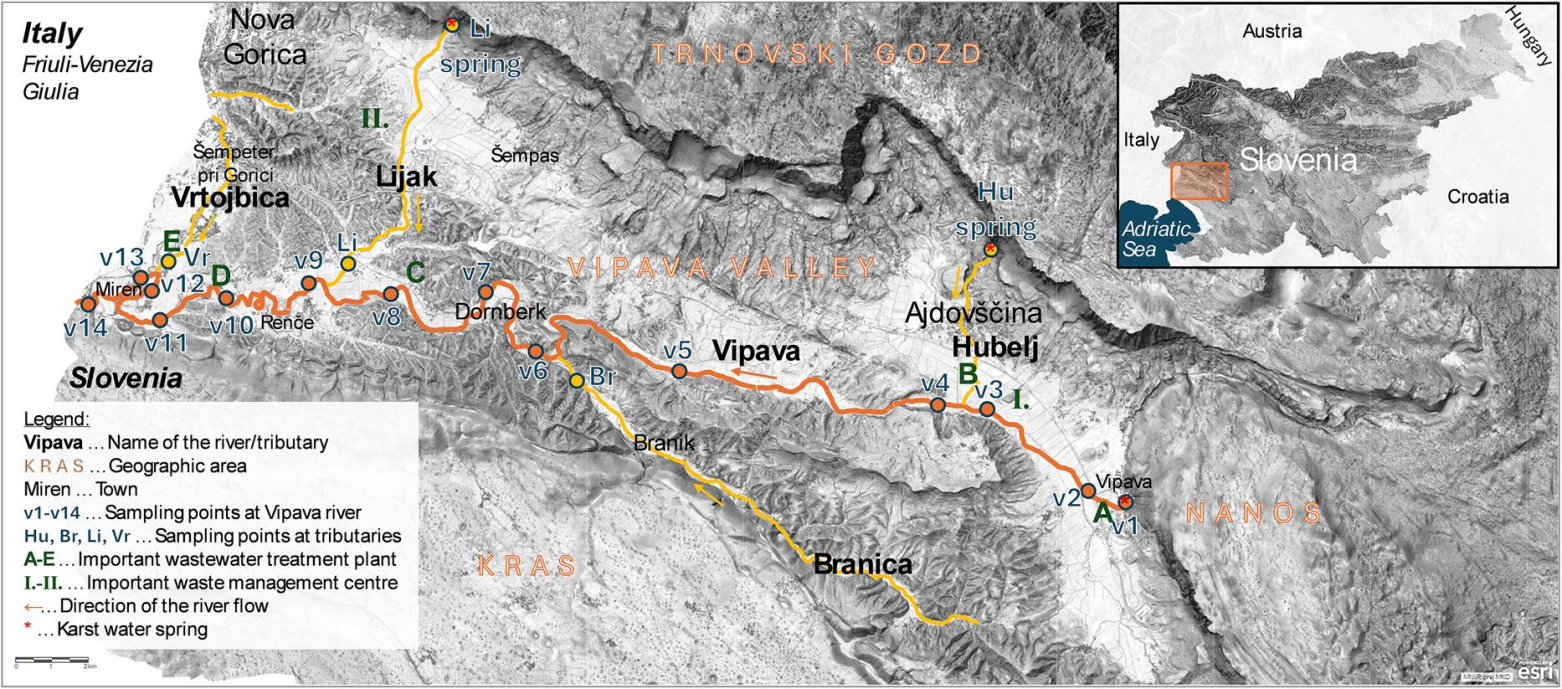
Geographical coverage, sampling locations, and studied waterways. The background maps were obtained from GeoHub-SI (2026) (with permission)

Grab sampling was conducted on 2 and 3 March 2024, at the locations shown in Fig. 1 and coordinates in Table SM2. Hydrological conditions are presented in Table SM3 and according to the data, water levels were within the 10-year average (Fig. SM3). One litre of water sample was collected in a Schott glass bottle, 2 m from the riverbank, from 0.5 m depth, using a retractable telescopic pole. The main water body was the Vipava river (samples V1−V14) with tributaries Lijak (spring and downstream part Li), Hubelj spring, Branica (Br), and Vrtojbica (Vr). Water samples had conductivity between 261 and 424 µS cm^‒1^, and pH ranged from 7.2 to 8.3 (Table SM2).

### Solid-phase extraction

The SPE procedure was previously developed by Kravos and Prosen (2024) but using simple water matrices. In the present study, the procedure was adapted for ultra-trace analysis of river water. Different 6-mL SPE cartridges were tested with 250-mL samples containing an initial CECs concentration 20 ng L^−1^ in MQ water: Oasis HLB (Waters, 200 mg, Wexford, Ireland), Supel Swift HLB (Supelco, 200 mg, Bellefonte, USA), Strata SDB-L (Phenomenex, 200 mg, Torrance, USA), and Supelclean LC-18 (Supelco, 1000 mg, Bellefonte, USA). The effect of increased salinity was evaluated by adding 3.8 wt.% NaCl (Merck, Darmstadt, Germany). The influence of sample volume was assessed with 250-mL, 350-mL, and 500-mL water sample. The latter two parameters were tested only with the Oasis HLB cartridge.

Adapted SPE procedure: Oasis HLB cartridge (6 mL/200 mg) was conditioned with 2 × 5 mL methanol, and equilibrated with 2 × 5 mL 0.1 wt.% HCl in MQ water (Supelco, Suprapur, Darmstadt, Germany). A 350-mL acidified water sample was passed through cartridge at a rate of one drop per second, followed by a wash step (2 × 5 mL 0.1 wt.% HCl in MQ water), drying under a vacuum pump, and gradient elution with 2 × 2.5 mL methanol, 2.5 mL 40 vol.% acetone in methanol, 2.5 mL acetone, and 2.5 mL ethyl acetate (both from Honeywell, HPLC grade, Seelze, Germany). The eluent was dried under a stream of nitrogen, and the dry residue was reconstituted with a reconstitution solvent (a mixture of 0.25 mL methanol and 0.1 mL MQ water). Finally, 0.35 mL of extract was filtered before analysis through a 0.45-µm nylon syringe filter (Chrom4, Germany), yielding approximately 0.25 mL for further analysis. SPE recoveries and repeatability were determined on *N* = 3 replicates using a 20 ng L^‒1^ spiked real river water V10, as it contained the lowest concentrations of analytes while being representative of the overall river water matrix (Fig. 1). The procedural blank was obtained by SPE of the conditioned Oasis HLB cartridge without the water sample load step.

### Instrumental analysis

Analysis was performed by LC-MS/MS using a Vanquish UHPLC System – TSQ Quantis MS/MS (Thermo Scientific, San Jose, USA), following a method adapted from Kravos and Prosen (2024) for CECs analysis and a separate method by Kravos et al. (2025) for TPs analysis. Method details are provided in Table SM4. Briefly, separation was carried out on a Kinetex F5 column (Phenomenex, 100 mm × 3.0 mm, 2.6 µm), with an injection volume of 2 μL, and MS/MS operated in scheduled MRM mode, with all transitions and settings detailed in Table SM4.

External calibration of LC-MS/MS was performed with a multistandard solution in reconstitution solvent in the range 0−75 µg L^−1^. The limit of detection (LOD) was either the instrumental detection limit (IDL), calculated from calibration lines (Kravos & Prosen, 2024), or the method detection limit (MDL), calculated as the concentration found in a procedural blank multiplied by a factor of 3. The higher value of IDL and MDL was accepted as LOD. The limit of quantification (LOQ) was calculated as 3 times the value of the LOD. Matrix effects (MEs) were calculated as the ratio between the calibration line slope constructed in river water extract and the slope constructed in a neat reconstitution solvent for a given analyte. Two sets of MEs were determined: separately for the upper river course and for the lower river course (Table SM5). The determined analyte concentrations were corrected for MEs. For TPs analysis, samples were analysed with LC-MS/MS (Kravos et al., 2025) and with LC-QTOF HRMS (Waters, Acquity Premier, Xevo G3 QTOF, Wilmslow, UK) using an Acquity Premier BEH C18 column (Waters, 50 mm × 2.1 mm, 1.7 µm), 2 μL injection volume, Lock Mass correction, and MS^E^ continuum scan mode. The final LC-QTOF method is specified in Table SM6.

For chemical fingerprinting, river water extracts were analysed using the previously described LC-MS/MS method (first paragraph of this section), operating in TIC scan mode (detailed method provided in Table SM4). TIC chromatograms were plotted within the *m/z* range 200−1200 Da (the range 340−350 Da was subtracted due to abnormally high background signal), normalised to the highest signal intensity (3.5 × 10^8^ in extract V13), and visually compared. The second parameter was the ‘overall TIC signal’, defined as the sum of all signal intensities within the plotted *m/z* range. The values ranged from 15.5 × 10^9^ to 58.0 × 10^9^ ion counts (Table SM2).

### Quality assurance/quality control

Multistandard and stock solutions were stored in the freezer (−20 °C) for a maximum of 3 weeks. The SPE procedure was checked for recoveries and repeatability using a spiked real river water sample to simulate actual conditions as accurately as possible. River water samples were kept in the freezer for a maximum of 1 week before processing. Separate glassware was reserved and used exclusively for these analyses to avoid any sources of cross-contamination. Calibration curves maintained a minimum *R*^2^ > 0.98. LC-MS/MS signal stability and response were checked with a 10 µg L^−1^ quality control multistandard analysed during each analysis batch. Needle washing within injections with 20 vol.% methanol in MQ was used to avoid carry-over. MEs were studied in detail by the previously mentioned procedure.

### Environmental risk assessment

Risk quotients (RQs) were calculated as the ratio *c*/PNEC, where *c* is the analytically determined analyte concentration and PNEC is the lowest predicted no-effect concentration. PNECs were retrieved from the NORMAN Ecotoxicology Database - Lowest PNECs (2025) as provided there. A RQ > 1 indicates an elevated environmental risk (Selak et al., 2022). Additionally, chronic toxicities to green algae, *Daphnia*, and fish were predicted using ECOSAR (US EPA) software, version 2.2 (2025). These values are calculated by the software as the geometric mean between the lowest-observed effect concentration (LOEC) and the no-observed effect concentration (NOEC).

## Results and discussion

### Method adaptation and performance

The selection of CECs included multiclass compounds with various chemical properties and uses (Table 1), which made method adaptation particularly challenging. Of the 18 initial target CECs, 4 analytes (CYC, AMA, MOX, and MBT) were excluded from further consideration due to low SPE recoveries (Table 1). The final SPE-LC-MS/MS method enabled single-injection quantitative analysis of 13 target analytes with sub-6 ng L^‒1^ LODs, SPE recoveries ranging from 17% (CAM) to 116% (RAM) (except 154% for NEO), and %RSDs below 14% (except 20% for THP) (Table 1). MEs were mostly below 40% (signal suppression), except where indicated otherwise in Table 1 (see the calculations in Table SM5). Abnormally high MEs were observed for the artificial sweetener NEO (*t*_R_ 11.2 min), in most cases reaching 150‒200% signal enhancement. The two remaining UV filters AVO and OCT were subjected to qualitative analysis within the same LC-MS/MS method (Table 1) due to high SPE %RSDs. The 42 suspect TPs were re-analysed with a qualitative approach and with a separate LC-MS/MS method due to the unavailability of standards.

**Table 1:** Overview of the analytes, their properties, and analytical method performance

Analyte	Classification	SPE performance^a^	LOD (ng L^‒1^)^b^		LOQ (ng L^‒1^)^c^	Matrix effect (%)^d^		PNEC (μg L^‒1^)^e^	Analysis
			MDL	IDL		Upper	Lower		
TBP	Fire retardant, plasticizer, additive	93 ± 4	1.0	0.25	3.0	−11	−24	24	Quantitative
THP	Fire retardant, plasticizer, additive	27 ± 20	5.9	1.2	18	−22	−46	(0.039)	Quantitative
ACS	Artificial sweetener	21 ± 10	n/f	0.80	2.4	−21	−19	(72.4)	Quantitative
CAM	Artificial sweetener	17 ± 6	n/f	3.5	11	−10	−18	(18.5)	Quantitative
NEO	Artificial sweetener	154 ± 4	0.54	0.11	1.5	180	168	(1.37)	Quantitative
TEL	Antihypertensive drug	71 ± 14	0.75	0.23	2.4	−15	−33	49	Quantitative
RAM	Antihypertensive drug	116 ± 1	1.0	0.15	3.0	−1	−12	1000	Quantitative
ROS	Antihyperlipidemic drug	87 ± 4	n/f	0.27	0.81	−13	−25	1.8	Quantitative
SIM	Antihyperlipidemic drug	49 ± 2	4.7	0.18	14	−23	−40	2.63	Quantitative
CAF	Stimulant, food additive	71 ± 2	2.0	0.43	6.0	26	9	1.2	Quantitative
PIR	Insecticide	103 ± 5	n/f	0.11	0.33	−23	−35	0.09	Quantitative
ACE	Insecticide	91 ± 3	4.4	0.21	13	−28	−36	0.037	Quantitative
DIF	Herbicide	54 ± 6	0.77	0.33	2.4	−34	−47	0.01	Quantitative
OCT*	UV filter	29 ± 49	46	1.6	138	−89	−49	0.27	Qualitative
AVO*	UV filter	30 ± 44	2.6	0.77	7.7	−19	−54	(0.12)	Qualitative
TPs**	42 suspect transformation products	n/a	n/a	n/a	n/a	n/a	n/a	n/a	Qualitative
MBT*	Anticorrosion agent, additive	< 10	n/f	0.65	1.9	−82	−39	4.1	Excluded
AMA*	Monomer, reagent, additive	< 10	n/f	0.39	1.2	n/a	n/a	(36.4)	Excluded
MOX*	Antibiotic	n/a	n/f	1.1	3.4	−51	162	0.13	Excluded
CYC*	Herbicide	< 10	0.52	0.23	1.5	−43	−43	464	Excluded

^a^Average value of the SPE recoveries with the respective %RSDs (*N* = 3); filtration losses are included in the overall values. ^b^The higher value (MDL or IDL) was selected for LOD. ^c^LOQ = 3 × LOD. ^d^Calculated for lower and upper river course. ^e^Predicted no-effect concentration (PNEC), retrieved from the NORMAN Ecotoxicology Database - Lowest PNECs (2025); values in brackets are predicted by QSAR and are not experimentally determined. *Concentration in a river water extract (μg L^−1^) is given. **Screened 4 MBT TPs, 6 CYC TPs, 8 ROS TPs, 3 TEL TPs, 10 PIR TPs, 1 DIF TP, 8 MOX TPs, and 2 AVO TPs; a list was adopted from Kravos et al. (2025). Notations: *n/a*, not assessed; *n/f*, no MRM transition signal found

**Fig. 2:**
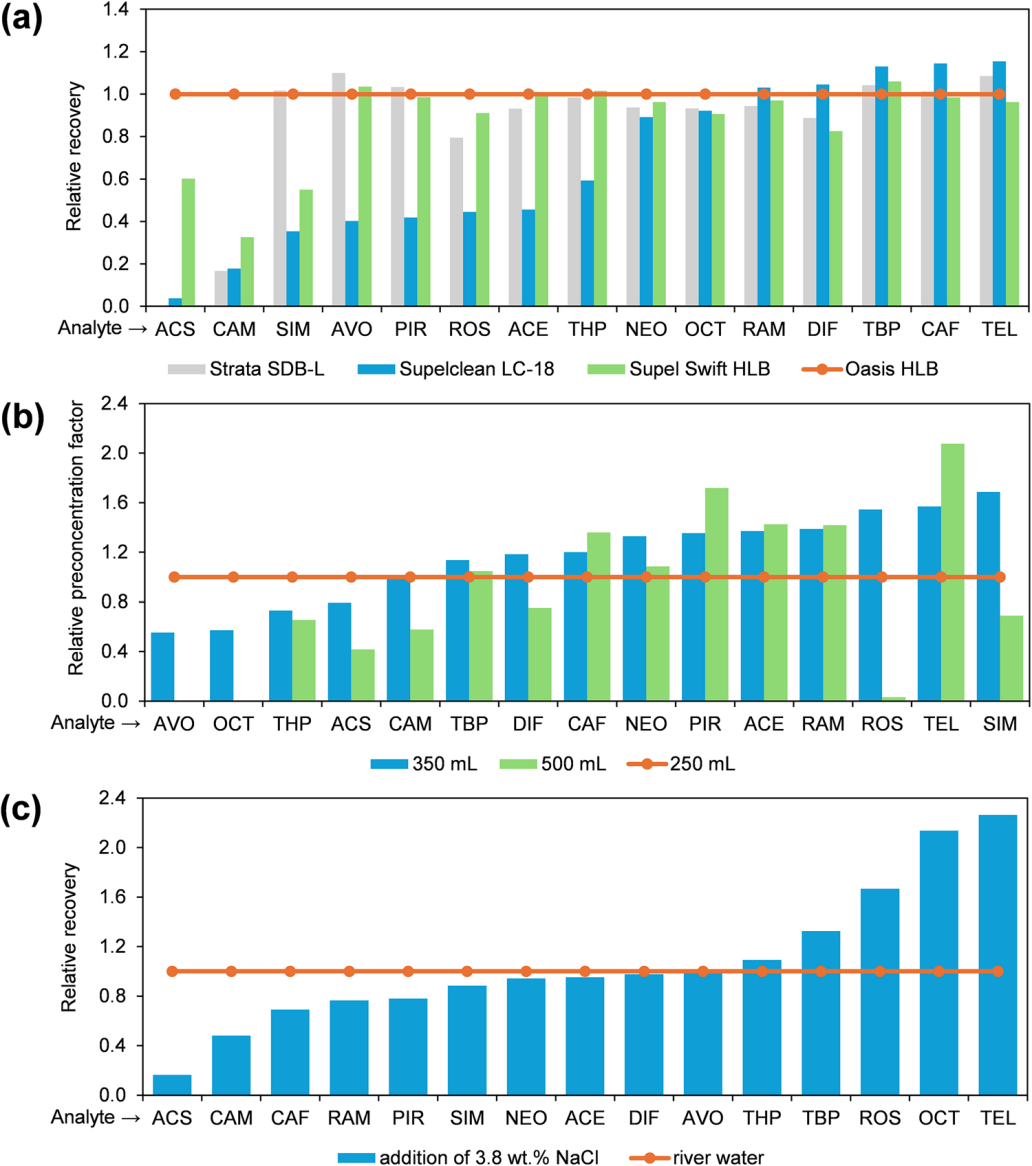
(**a**) Screening of the cartridges, (**b**) influence of sample volume (Oasis HLB cartridge only), and (**c**) influence of ionic strength on SPE performance (Oasis HLB only). All relative parameters on the *y*-axis are normalised to the values indicated by the orange horizontal line. Note that the SDB-L and HLB cartridges contained 200 mg, while the C18 cartridge contained 1000 mg of sorbent. The preconcentration factor in graph **b** was calculated as the ratio of water sample volume to SPE extract volume, multiplied by recovery

The chemical nature of the SPE sorbent is crucial in method development. There is a wide selection of specific SPE sorbents tailored to the chemical structure of analyte, sample volume, and type of matrices. The hydrophilic-lipophilic balanced (HLB) sorbent, chemically known as *N*-vinylpyrrolidone-divinylbenzene and commercially available as Oasis HLB by Waters, Strata X by Phenomenex, and Supel Swift HLB by Supelco, remains the first choice for multi-residue analysis (Kravos & Prosen, 2024) and normally using acidic pHs (García-Córcoles et al., 2019; Sadutto & Picó, 2020). In the present study, the HLB sorbent again demonstrated its versatility and high efficiency compared to octadecyl (Supelclean LC-18) and styrene-divinylbenzene sorbent (Strata SDB-L) (Fig. 2a). There were only minor differences between the two commercial variants of the HLB cartridges. Oasis HLB provided higher recoveries for very polar artificial sweeteners (ACS, CAM), consistent with literature reports (Arbeláez et al., 2015; Gvozdić et al., 2020), compared to Supel Swift HLB. In the final stage, a 1000-mg C18 cartridge was also tested, but it did not reach improvements in recoveries, showing low recoveries for polar and, interestingly, also for some non-polar analytes (Fig. 2a).

Increasing the sample volume raises the preconcentration factors and thus lowers the LODs. However, an excessively high sample volume can decrease extraction efficiency (Sadutto & Picó, 2020), increase MEs (Arbeláez et al., 2015), and increase the chance for cartridge clogging. Using Oasis HLB, the initial 250 mL volume was optimal only for very polar (ACS, CAM) and non-polar analytes (OCT, AVO, THP), but there was room for improvement for other analytes (Fig. 2b). Artificial sweeteners have already been shown to have recoveries highly influenced by sample volume. For instance, recoveries for ACS and CAM rapidly decreased from over 70% to below 30% when increasing the sample volume from 50 to 200 mL (Gvozdić et al., 2020), which aligns with our observations. However, using a sub-100 mL sample volume solely to achieve better preconcentration of ACS and CAM would not be beneficial overall for other analytes. Given all the analytes, preconcentration factors increased optimally from 250 to 350 mL (Fig. 2b). However, a further increase to 500 mL had a negative impact on other analytes, such as DIF, ROS, SIM, and NEO, due to reaching breakthrough volumes, so a volume of 350 mL was selected as optimal. Lastly, ionic strength can be highly important for SPE efficiency for some CECs (Liu et al., 2004), but not for other CECs (Liu et al., 2009). Increased salinity may affect the bonding affinity of CECs to the sorbent and is associated with the salting-out effect, depending on the type of interaction and water solubility of CEC. Using Oasis HLB, ionic strength was in the present study increased by adding NaCl to the river water matrix. SPE efficiency decreased (Fig. 2c), demonstrating NaCl’s interfering effects particularly to retention of very polar ACS and CAM.

The adapted SPE method used an acidified 350-mL water sample and a 200-mg Oasis HLB cartridge, which provided a wide range of recoveries (Table 1). Recoveries below 50% were observed for very polar (ACS, CAM) and non-polar analytes (THP, OCT, AVO). An additional systematic multivariate optimisation approach, such as factorial design (Lukić et al., 2022), could potentially provide better SPE conditions with higher overall recoveries. However, this was not tested in the present study. Very polar artificial sweeteners are problematic analytes that usually exhibit lower SPE recoveries when using generic sorbents, such as Oasis HLB (Arbeláez et al., 2015). The use of more specific ion-exchanging sorbents may be a better option for these analytes (Gvozdić et al., 2020). Recoveries for the remaining polar and semi-polar analytes were above 50% (Table 1). The %RSDs were acceptable for most analytes, except for OCT and AVO. In comparison, high recoveries and acceptable %RSDs were obtained for these two analytes by Bratkovics and Sapozhnikova (2011) using a 200-mL water acidified sample and a 500-mg Oasis HLB cartridge.

The analytes AMA, CYC, MOX, and MBT were excluded from the study (Table 1). The null recoveries of AMA and CYC could be explained by their very high polarity and susceptibility to acid hydrolysis (Monadjemi et al., 2013), respectively. AMA was the most polar analyte in the present study and would also require ion-exchange sorbents or multilayered SPE with self-packed cartridges containing various sorbents (Neuwald et al., 2021). MOX tended to adsorb onto surfaces and filters, resulting in high fluctuations in recoveries. The null recovery of MBT could be attributed to its tendency to oxidise to bis-(2-benzothiazolyl)-disulfide, as observed by Kloepfer et al. (2004). Authors resolved this by adding the reducing agent glutathione, which significantly increased the recovery. In the present study, SPE of MBT aqueous solution was also performed in the presence of 300 μM glutathione. The suspected oxidised product, bis-(2-benzothiazolyl)-disulfide, was not identified by LC-MS/MS, but MBT recovery increased from null to 93%, supporting the above assumptions.

### Occurrence of CECs

The SPE-LC-MS/MS method enabled analysis of CECs in a well-defined sub-Adriatic river system. Numerous sampling sites were concentrated in one region, which allowed for a better understanding of the river’s dynamics and identification of the sources of anthropogenic pressure (Köck-Schulmeyer et al., 2021). However, long-term monitoring would be needed to assess temporal variability of contamination, the influence of droughts and floods, and the occurrence of other types of CECs.

A map and a panoramic photo of the region are shown in Fig. 1 and Fig. SM7, respectively. The studied sub-Adriatic region is located in the western Slovenia and is surrounded by karst plateau Kras to the south and parts of the Dinaric plateaus (Trnovski gozd and Nanos) at the northern and eastern edges. The region’s western edge opens onto the plains of Friuli-Venezia Giulia region in Italy. The Vipava river, known as Vipacco in Italian, is a 49-km-long Slovenian-Italian river and the main water body of the region with a catchment area of approximately 590 km^2^. Its waters flow over Eocene flysch deposits and feed one of the few alluvial aquifers in the Slovenian parts of the Adriatic Sea catchment. The river flows through the region from its spring in Vipava town downstream to its confluence with Soča (Isonzo) river in Italy (Fig. 1). Soča river flows directly into the Gulf of Trieste, North Adriatic Sea, only approximately 30 km downstream of the Vipava-Soča confluence in Italy (Brečko Grubar & Kovačič, 2010; Jelovčan & Šraj, 2022). The Slovenian part of the studied region has approximately 70,000 inhabitants (data from the Statistical Office of Republic of Slovenia, 2025), two big WWTPs, Nova Gorica central WWTP (50,500 population units) and Ajdovščina WWTP (42,000 population units), scattered and non-intensive agricultural activity, as well as two big landfills and waste collection and management centres near Ajdovščina and Nova Gorica (see Fig. 1).

Water sources (Vipava, Hubelj, Lijak springs) beneath the karst aquifer of Trnovski gozd at the northern edge of the Vipava valley did not contain the target CECs, except for CAF, which was found at concentrations around the LOQ (Table SM8). This is particularly valuable information from a public health perspective, as the Hubelj spring is used for drinking water production in the region. Karst groundwaters are especially vulnerable to indirect contamination from other interconnected water sources and may therefore contain other CECs. Some have been monitored in freshwater from part of the Dinarides along the Adriatic coast, in Croatia (Selak et al., 2022, 2024). It has also been shown that CECs, especially those with hydrophilic properties, may not be removed by drinking water treatment and can therefore easily enter drinking water from their source groundwater (Papagiannaki et al., 2021).

**Fig. 3:**
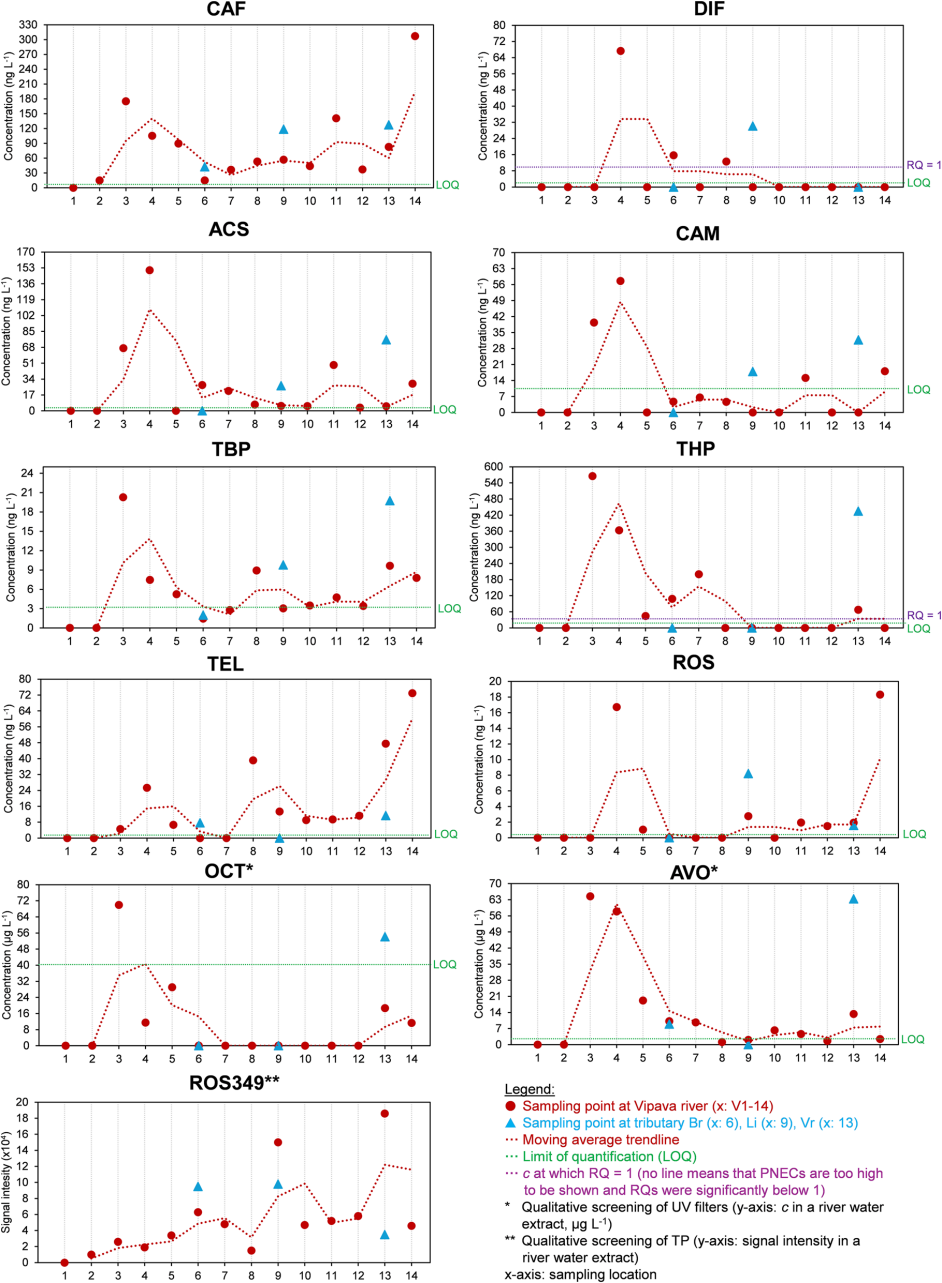
Distribution of CECs and TP ROS349 in the Vipava river system with marked RQs (where applicable)

The distribution of frequently detected CECs is shown in Fig. 3, with exact numbers shown in Table SM8, and a comparison with the literature provided in Table 2. Analytes ACE, PIR (occasionally detected below LOQ), SIM, RAM, and NEO were generally not found in any of the samples (Table SM8). CAF was the most common analyte found in water samples with concentrations often exceeding 50 ng L^‒1^. The occurrence trend in Fig. 3 indicates constant and scattered influxes of CAF, with concentrations increasing near WWTPs, for example, in the river stretches V3−V5 and later in V13−V14. CAF can be considered an anthropogenic tracer, as it is one of the most widely consumed substances, resulting in high emissions into the environment (Papagiannaki et al., 2021). CAF is highly biodegradable in the environment and in conventional WWTPs (Škrbić et al., 2018), but its high influx of CAF into WWTPs and its hydrophilicity reduce its removal efficiency (Gracia-Lor et al., 2017). In the literature, CAF has been reported to be frequently found in freshwater at concentrations below 10 ng L^‒1^ (Klančar et al., 2018; Papagiannaki et al., 2021), but also up to several µg L^‒1^ (Česen et al., 2019; Škrbić et al., 2018). CAF has even been detected in groundwater in Slovenia. For instance, Koroša et al. (2016) determined a median concentration of 1.0 ng L^‒1^ of CAF in Slovenian groundwater in Maribor.

**Table 2:** Published results on the target CECs determined in the wider geographical region

Reference*	CEC	Min. and max. concentration (ng L^−1^)	Positive vs. all samples	Water body and region
Gvozdić et al., 2020	ACS CAM NEO	19−47 66 27	5/5 1/5 1/5	Sava and Danube rivers in North Serbia
Pantelaki and Voutsa, 2021	TBP THP	< 11418 < 1568	22/24 19/24	Rivers and urban streams in North Greece
Loos et al., 2017	TBP	< 93	45/71	Danube river system
Németh et al., 2025	OCT AVO	142 159	1/1 1/1	Lake Balaton in Hungary
Zulfiqar et al., 2026	OCT	74−23100 (seawater)	14/14	Adriatic coast in Central Italy
Česen et al., 2019	CAF	37−1390	6/7	Sava river in Slovenia and Croatia
Klančar et al., 2018	ROS CAF	2.8 1−3	1/6 6/6	Rivers and lakes in Central and Eastern Slovenia
Anagnostopoulpou et al., 2022	CAF TBP THP TEL RAM PIR ACE	< 208 < 17 < LOQ 3, 13.5 n/d n/d n/d	18/18 10/18 1/18 2/18 0/18 0/18 0/18	Lakes in Central and North Greece
Tauchnitz et al., 2020	DIF DIF PIR ACE	< 17000 (grab sample) 3−6 (composite sample) n/d n/d	46/108 4/118 0/108 0/108	Surface waters in Central Germany

*Limited to recent studies from the wider Adriatic region or (if no data available) from the closest surrounding European region. Notations: *n/d* not detected, *LOQ* limit of quantification

Most analytes showed similar occurrence trends as their concentrations notably increased in the river stretch V3−V4 in the upper Vipava valley (Fig. 3), suggesting possible emissions from Ajdovščina WWTP and/or landfill; however, further studies are needed to confirm this general suspicion. CECs were also present at high concentrations in the receiving water Vr from Nova Gorica WWTP, but not as much in the secondary receiving Vipava river stretch V13−V14. High concentrations of artificial sweeteners ACS and CAM in the river stretch V3−V4 may be correlated with the vicinity of the prominent food-processing industry in Ajdovščina (Fig. 3). Their presence in freshwaters has been observed previously (Arbeláez et al., 2015), and they have even been detected in spring water from karst aquifers (Selak et al., 2022), as well as elsewhere (Table 2). Another artificial sweetener, NEO, was not detected. Organophosphates (TBP, THP) and UV filters (OCT, AVO) were also found at low abundancies, except again in V3−V4 and Vr, all of which appear to be directly influenced by WWTPs and/or landfill (Fig. 3). Organophosphates are rarely reported in the literature. Here, we detected relatively high concentrations, which probably originated from Ajdovščina landfill, as their concentrations were highest in the river stretch V3 (Fig. 3); however, it is also possible that this was a one-time event or that they came from other accidental sources. The presence of UV filters was expected (Fig. 3), as they are ingredients in commonly used cosmetic products such as sunscreens, and these CECs eventually end up in wastewaters and thus in freshwaters (Gago-Ferrero et al., 2011). The occurrence of other UV filters was investigated by Česen et al. (2019), who reported very high concentrations and detection frequencies of 4-hydroxybenzophenone and 2-hydroxy-4-methoxybenzophenone (oxybenzone) in Slovenian wastewaters and freshwaters. Other relevant studies are shown in Table 2.

Pharmaceuticals are intentionally developed to influence organisms’ physiological functions at very low concentrations, making them particularly problematic CECs, especially for small freshwater organisms (Gavrilescu et al., 2015). In the present study, the antihypertensive drug TEL and the antihyperlipidemic drug ROS were found above the LOQ (Fig. 3). Their presence appears closely linked to the vicinity of WWTPs since their concentrations were highest in the river stretches V3−V4, V8−V9, and V13−V14. However, it is surprising that concentrations were low in the primary receiving water (tributary Vr) from Nova Gorica WWTP and high in the nearby Vipava river stretch V14 (Fig. 3). ROS also showed a high concentration in the tributary Lijak (site Li, Fig. 3), which could indicate the impact of Nova Gorica landfill and/or the influence of domestic sewage systems – a matter requiring further research. ROS was found at lower concentrations compared to TEL, which might be explained by ROS’s lower (photo)stability, leading to the formation of numerous TPs (Kravos et al., 2025) that were also detected in the present study. TEL and ROS are widely used drugs in Slovenia, with approximately 132,000 and 723,000 prescriptions in 2023 for TEL (trade names Tolura, Tolucombi) and ROS containing medicines (trade names Sorvasta, Coupet), respectively (data from The Health Insurance Institute of Slovenia, 2025). Sartans have also been detected in other regional studies (Table 2) and in other Slovenian surface waters. For instance, Klančar et al. (2018) reported a high occurrence of irbesartan (up to 9.3 ng L^−1^) and valsartan (up to 47.2 ng L^−1^), both appearing above the LOQ in all Slovenian lakes and rivers. In contrast, ROS was mostly below the LOQ (Klančar et al., 2018) (Table 2) while in the present study it often exceeded its LOQ (Fig. 3). The other two pharmaceuticals, SIM and RAM, were not detected. RAM is rarely found in freshwaters as it is probably hydrolysed into the structural analogue ramiprilat (Ghoshdastidar et al., 2015; Giebułtowicz et al., 2016), while SIM is rarely analysed. To date, there have been some reports of other pharmaceuticals detected in Slovenian waters. For instance, Kosjek et al. (2005) quantified naproxen and diclofenac in rivers Krka, Ljubljanica, Sava, Mura, Drava, and Pšata. Diclofenac was also frequently detected by Česen et al. (2018) in Slovenian wastewaters and receiving waters. In the Adriatic region, Grobin et al. (2024) recently found cumulative concentrations of 1.2 ng L^‒1^ and 2.9 ng L^‒1^ of natural hormones, bisphenols, and synthetic steroids in Hubelj and Vipava, respectively, with estradiol as the predominant analyte.

Among the pesticides, only the herbicide DIF was exceeding LOQ in the present study. ACE and PIR were rarely detected CECs in other regional freshwaters (Table 2). DIF was found in river stretches V4, V6, V8, and in tributary Li, where concentrations were above the LOQ but reached only a few ng L^‒1^, with a maximum of approximately 67 ng L^‒1^ (Fig. 3). The sources may have been the cultivation of winter cereals using crop protection sprayers during the winter season in agricultural areas of the region (noting that sampling took place in early March). For instance, plant protection products such as Alliance (Nufarm) have a high content of DIF and are approved for use in Slovenia (Administration for Food Safety, Veterinary Sector and Plant Protection - A list of registered phytopharmacutical agents in Slovenia, 2025). DIF is known to be a moderately persistent herbicide with a half-life of 76 days (microbial degradation). It also undergoes strong sorption onto suspended particles and may therefore be transported over long distances, making it difficult to link DIF occurrence to its sources (Tauchnitz et al., 2020). For instance, Barbieri et al. (2020) determined DIF in the agriculture-impacted Llobregat river, Spain, at concentrations ranging between 130 and 150 ng L^‒1^. Recently, Peris et al. (2024) found DIF in water samples and sediments in two natural parks in Spain, highlighting its potential risks to aquatic organisms. Tauchnitz et al. (2020) also frequently detected DIF up to a few µg L^‒1^ in German surface waters (Table 2). Pesticides in general have been detected in Slovenian natural waters. For instance, Koroša et al. (2016) found desethyl-atrazine, atrazine, and desethyl-terbutylazine in groundwater in Maribor and the river Drava.

### Occurrence of transformation products

**Fig. 4:**
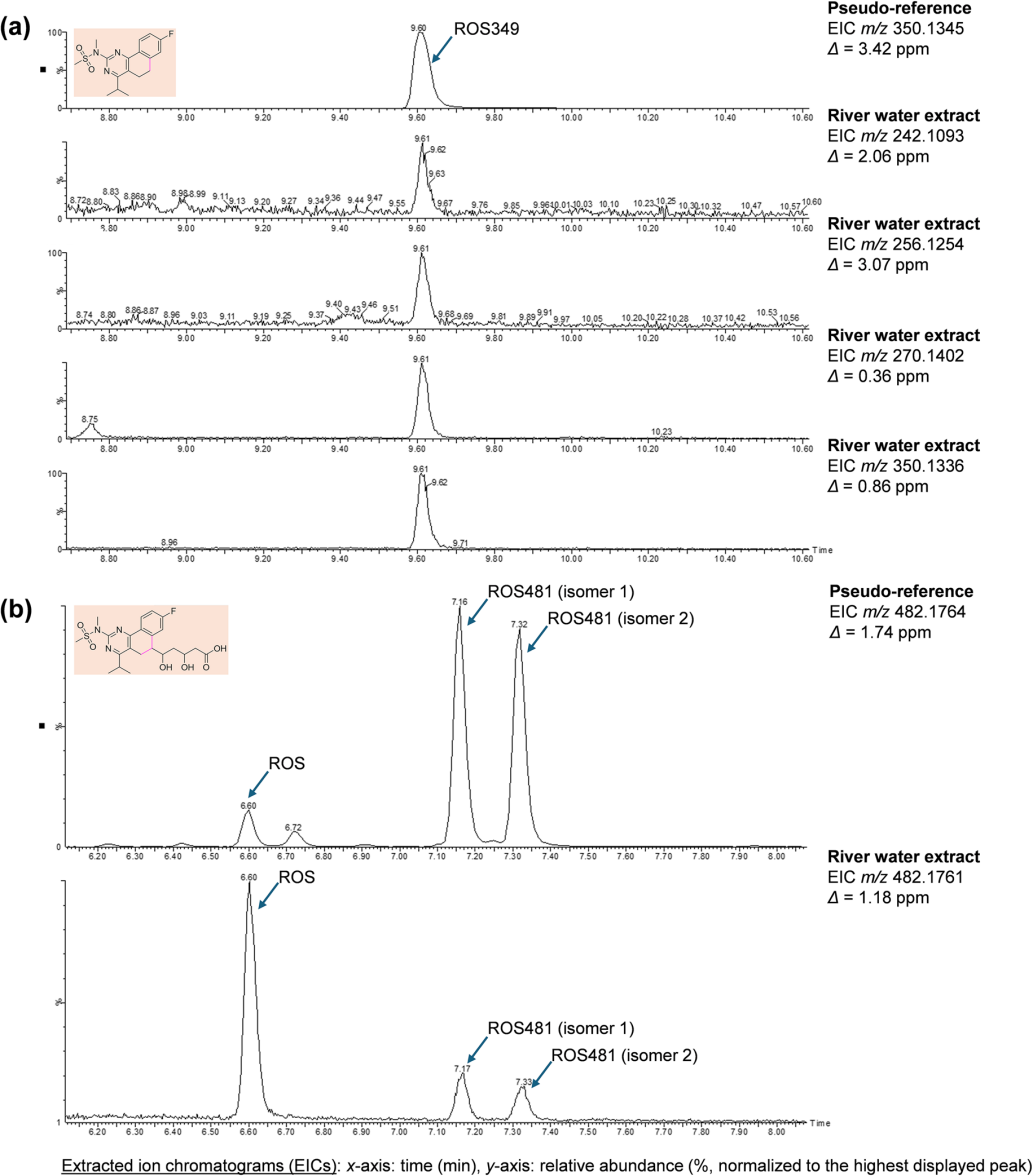
Identification of (**a**) ROS349 (*t*_R_ = 9.60 min) and (**b**) ROS481 TPs (*t*_R_ = 7.16, 7.32 min) in river water extract, and alignment of the peak with a pseudo-reference peak using LC-QTOF HRMS. Only the precursor (molecular ion) of ROS481 was observed in section **b**; ROS is isobaric with ROS481, so its peak also appears in the EICs

Screening of TPs is rarely reported in the literature, so the inclusion of TPs in the present study adds additional novelty and improved knowledge on CECs (Kravos & Prosen, 2024; Maculewicz et al., 2022). However, quantification of TPs remains largely impossible due to the commercial unavailability of the standards, so they are sometimes custom-synthesised (Česen et al., 2018). One of the first studies on this topic in Slovenia was conducted by Česen et al. (2018, 2019), who screened for several known TPs of selected pharmaceuticals, such as diclofenac, and by Gornik et al. (2020, 2021), who analysed paroxetine TPs and sertraline TPs in surface waters. Using knowledge of TPs’ fragmentation patterns, 42 suspect TPs were analysed in the present study by LC-MS/MS operating in MRM mode as the first level of confirmation (Fig. SM9), which is rarely reported for suspect screening. Identification confirmation was then followed by high-resolution mass spectrometry to increase identification confidence to the second level (Fig. 4 and Fig. SM10).

ROS349 and ROS481 were the only two TPs identified by LC-MS/MS (Fig. SM9 and Fig. SM10) and confirmed by LC-QTOF in river water extracts (Fig. 4). ROS TPs were previously identified also by Astarita et al. (2007) and Kravos et al. (2025) during laboratory degradation of ROS. ROS349 has only recently been identified in the actual freshwater by LC-MS/MS (Kravos et al., 2025); however, no distribution analysis, nor confirmation with HRMS, was performed. The confidence level for identification of ROS349 in the current study was high since all four screened MRM transitions were confirmed with comparable ion ratios, and there was alignment of the retention time with a reference peak (Fig. SM9). Precursor and fragment ions were also observed with LC-QTOF, with mass accuracy below 5 ppm (Fig. 4 and Fig. SM10). ROS349 was frequently identified, suggesting its omnipresence in the Vipava river system (Fig. 3). Its presence did not correlate with ROS occurrence (Fig. 3). The highest abundance was observed at the secondary receiving water V13 site, which could be explained by the vicinity of WWTP as ROS349 might be by-product of water treatment processes, such as ozonation or UV irradiation (Astarita et al., 2007). Regarding the second TP, ROS481, the peak also aligned with a reference peak, but only two MRM transitions were confirmed by LC-MS/MS (Fig. SM9), and only the precursor ion was observed by LC-QTOF, which overall decreases the identification confidence level (Fig. SM10).

### Environmental risk assessment

The presence of CECs in the natural environment may have consequences; CECs may cause unintended negative effects on aquatic organisms, such as histological abnormalities and impacts on reproduction (Gavrilescu et al., 2015). In addition, the adverse effects of CECs on human health remain largely unknown, raising many possible concerns. Therefore, environmental risk assessment is becoming increasingly important in such studies and is based on the calculation of RQs (see the “Environmental risk assessment” section in 'Materials and methods'), where RQ > 1 is accepted as a threshold for possible ecological hazard. This approach has been used in similar studies (Česen et al., 2018, 2019; Grobin et al., 2024; Selak et al., 2022; Verovšek et al., 2023), although experimental toxicological testing has also been reported in the literature, such as the algal growth inhibition test (Verovšek et al., 2023).

The RQ = 1 values were directly labelled in Fig. 3 where applicable and RQ ranges are shown in Table SM8. Most of the quantified CECs in the present study showed RQs < 1 due to low concentrations and/or high PNECs, indicating a low ecotoxicological risk (Table SM8). The exceptions were THP and DIF due to RQs > 1 at several sampling sites (Fig. 3 and Table SM8), particularly for THP at V3, V4, and Vr. Site Vr, the receiving water of Nova Gorica WWTP, was rapidly diluted by the Vipava river at V13, which did not show any elevated ecological risks from THP. In contrast, the high concentration of THP in the river stretch V3−V4 in the upper valley, Ajdovščina region, may pose some environmental concerns; especially because Vipava river is part of Natura 2000, a known habitat for the endangered Italian agile frog (*Rana latastei*) (Natura 2000 in Slovenia, 2025). However, long-term monitoring would be necessary to fully confirm this claim.

As for the two ROS TPs, they were not quantified, and PNECs for these two compounds are still unavailable, so RQs were not determined. However, attempts were made to predict their chronic toxicities. ROS349 was predicted by ECOSAR software to have much higher chronic toxicity to green algae (959 µg L^‒1^), *Daphnia* sp. (441 µg L^‒1^), and fish (75 µg L^‒1^) compared to ROS. Therefore, the occurrence of ROS349 in the Vipava river at V13 and V9 may pose a certain risk; however, knowledge gaps and the absence of experimental data limit further discussion.

### Chemical fingerprinting

**Fig. 5:**
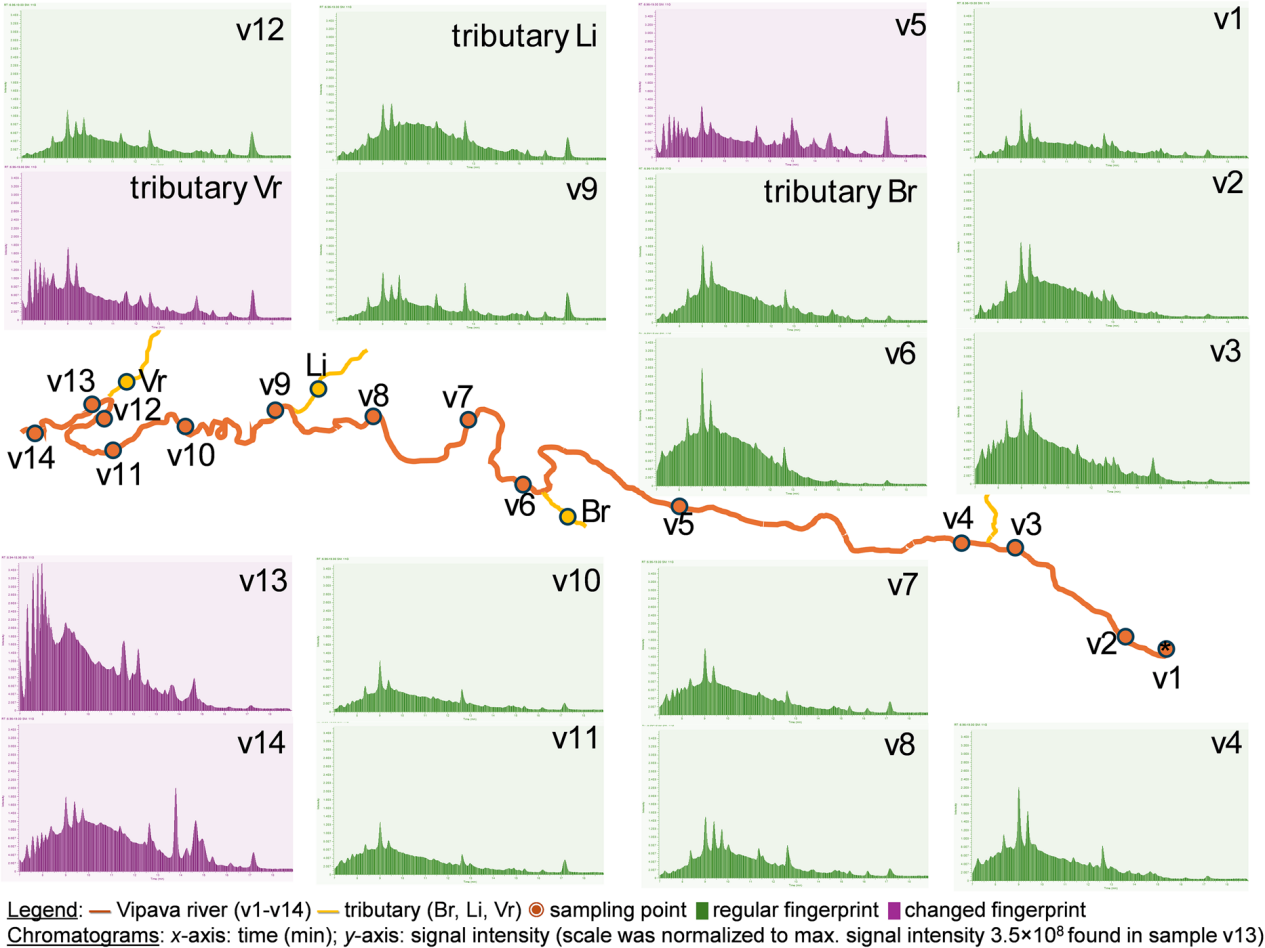
Changes in TIC LC-MS chromatograms of river water extracts along the river course

The composition of background substances may be characteristic of a particular water type. Any influx of tributary water or wastewater can influence the river’s natural chemical composition by introducing various anthropogenic compounds, including CECs and macro-contaminants. These compounds are largely extracted into river water extracts. Although SPE, like other extraction techniques, may exclude some group of compounds present in river samples, the river water extract still provides valuable overall information. Fingerprinting water samples with LC-MS can provide data on the occurrence of target, suspect (Lopez-Herguedas et al., 2022), and non-target micro-contaminants (Troxell et al., 2024; Xia et al., 2024), depending on the LC-MS acquisition mode. Another option is to consider the abundant, non-specific background compounds. This approach has rarely been reported in the literature but could be very useful for tracking anthropogenic or natural changes in the chemical properties of water along the river course, as well as for assessing water complexity and hydrodynamics. In the present study, TIC LC-MS chromatograms and the overall TIC signal (see the “Instrumental analysis” section) of river water extracts were used as qualitative descriptors for the water’s chemical fingerprint (Fig. 5).

Most background substances were eluted between the 8th and 11th minute of the analysis runtime (Fig. 5). The overall TIC signal varied between samples (Table SM2), but the shape was consistent, suggesting a uniform composition of samples along the river course (green chromatograms in Fig. 5). The tributaries also exhibited a fingerprint largely similar to those of the main Vipava river. However, some sites showed noticeable changes in the regular fingerprint. Additional peaks appeared and signal intensities increased for V5, Vr, V13, and V14 (purple chromatograms in Fig. 5). All these samples could be linked to wastewater influxes, especially V13 and Vr near Nova Gorica WWT, which aligns with previous results on CECs’ occurrence. Sample V12 displayed a regular fingerprint, but once the WWTP effluent from Vr merged with the Vipava river at sampling site V13, the fingerprint changed significantly. The original fingerprint was almost restored at sampling site V14, a few kilometres downstream from V13. A further comparison of chromatograms for primary receiving water Vr and secondary receiving water V13 supports the assumption that the background substances may have originated from the WWTP effluent. Interestingly, such alterations in TIC LC-MS chromatograms were not observed for the river stretch V3−V4 near Ajdovščina WWTP and/or landfill in the upper Vipava valley, where CEC concentrations were highest (compare with Fig. 3). Beyond visual comparison of data, applying multivariate computational analysis or machine-learning techniques could provide deeper insights and uncover additional correlations in the future.

## Conclusions

The continual development of ultra-sensitive analytical procedures based on SPE-LC-MS/MS is essential for the analysis, study, and control of CECs in various water matrices. Knowledge of the occurrence of CECs in Slovenian natural waters remains unclear despite Slovenia’s unique geographical and hydrological position in Europe. Adriatic and sub-Adriatic rivers, groundwater from karst landscapes (such as the Dinarides), and seawater from the Gulf of Trieste in the North Adriatic Sea are particularly under-researched. The present study shows SPE method adaptation, the occurrence and distribution of CECs and TPs, environmental risk assessment, and chemical fingerprinting to track anthropogenic stress in the Slovenian-Italian Vipava river system. The SPE-LC-MS/MS method followed a generic procedure using Oasis HLB and 350 mL of acidified water sample. The method enabled quantitative analysis of 13 CECs, and qualitative analysis of 2 UV filters, while 4 initially included CECs were excluded due to performance issues. Separate LC-MS/MS methods were applied to the same river water extracts to perform screening of 42 suspect TPs in MRM mode and chemical fingerprinting in TIC scan mode to obtain TIC chromatograms (so-called river water fingerprints).

Results indicate limited anthropogenic contamination, which mostly correlated with the vicinity of regional WWTPs and landfills, and partially aligned also with chemical fingerprinting. The latter enables investigation of changes in the chemical composition of water downstream due to inflows from wastewater and tributaries, and may aid understanding of the river’s hydrodynamics and mixing events. CAF was detected in all analysed water samples. Artificial sweeteners, organophosphates, and UV filters occasionally reached high concentrations in the upper valley Ajdovščina region, likely influenced by local WWTP and/or landfill, and in the primary receiving water from the Nova Gorica WWTP, though further analyses are needed for confirmation. THP and DIF showed elevated risk quotients, indicating possible negative ecological effects. TEL and ROS were the two most frequently detected pharmaceuticals. Additionally, two rarely reported TPs, ROS349 and ROS481, were identified in river samples. ROS349 showed a distinct distribution profile along the river course and possible ecotoxicological concerns. Overall, several target CECs were detected for the first time in Slovenian freshwaters, along with TPs. This expands knowledge of CECs in parts of the sub-Adriatic region and highlights the need for long-term monitoring and multi-residue analyses of a larger set of contaminants in the future.

## Supplementary Information

Below is the link to the electronic supplementary material.

ESM 1 Supplementary Material 1 (PDF 1.16 MB)

## Data Availability

Data will be made available on reasonable request.

## Abbreviations

AMA: 2-acrylamido-2-methyl-1-propanesulfonic acid
MBT: 2-mercaptobenzothiazole
ACE: Acetamiprid
ACS: Acesulfame
AVO: Avobenzone (also known as butyl methoxydibenzoyl methane)
CAF: Caffeine
CEC: Contaminant of emerging concern
CAM: Cyclamate
CYC: Cycloxydim
DIF: Diflufenican
ESI: Electrospray ionization
EIC: Extracted ion chromatogram
HRMS: High-resolution mass spectrometry
HLB: Hydrophilic-lipophilic balanced
IDL: Instrumental detection limit
LOD: Limit of detection
LOQ: Limit of quantification
LC: Liquid chromatography
ME: Matrix effect
MDL: Method detection limit
MOX: Moxifloxacin
MRM: Multiple reaction monitoring
NEO: Neotame
OCT: Octocrylene
PIR: Pirimicarb
PNEC: Predicted no-effect concentration
QTOF: Quadrupole coupled to time-of-flight mass analyser
RAM: Ramipril
%RSD: Relative standard deviation expressed in percentage
RQ: Risk quotient
ROS: Rosuvastatin
SIM: Simvastatin
SPE: Solid-phase extraction
TEL: Telmisartan
TIC: Total-ion current
TOC: Total organic carbon
MS/MS: Triple-quadrupole mass analyser (also tandem mass spectrometry)
TP: Transformation product
TBP: Tris(2-butoxyethyl) phosphate
THP: Tris(2-ethylhexyl) phosphate
MQ: Ultra-pure water
WWTP: Wastewater treatment plant
