# Supplementary material for: Application of LC-MS/MS methods and risk assessment for selected emerging contaminants and their transformation products in a sub-Adriatic river system
Source: Environ Monit Assess. 2026 Jun 4;198(7):683. doi: 10.1007/s10661-026-15437-4 (PMC13236757; doi:10.1007/s10661-026-15437-4)

Supplementary Materials

Application of LC-MS/MS methods and risk assessment for selected emerging contaminants and their transformation products in a sub-Adriatic river system

Aleksander Kravos* and Helena Prosen

University of Ljubljana, Faculty of Chemistry and Chemical Technology, Večna pot 113, SI-1000, Ljubljana, Slovenia
*Corresponding author: aleksander.kravos@fkkt.uni-lj.si

SM1, Overview of the target CECs

**Ramipril (RAM)**<br>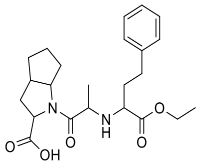<br>Certified reference material, Sigma Aldrich (China)	**Telmisartan (TEL)**<br>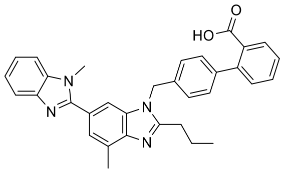<br>> 98.0%, TCI (Japan)	**Rosuvastatin (ROS)**<br>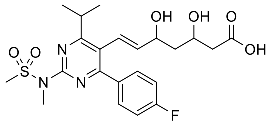<br>*Sodium salt*, > 97%, Thermo Fisher Scientific (China)	**Simvastatin (SIM)**<br>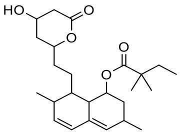<br>> 98%, Thermo Fisher Scientific (China)
**Cycloxydim (CYC)**<br>***excluded***<br>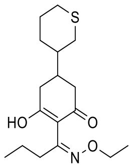<br>≥ 95.0% analytical standard, Pestanal – Sigma Aldrich (Switzerland)	**Diflufenican (DIF)**<br>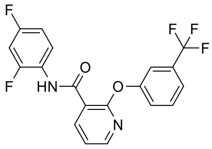<br>≥ 95.0%, analytical standard, Pestanal – Sigma Aldrich (Switzerland)	**Pirimicarb (PIR)**<br>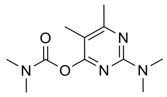<br>≥ 98.0%, Pestanal – Sigma Aldrich (Switzerland)	**Acetamiprid (ACE)**<br>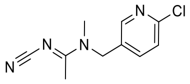<br>Certified reference material, Honeywell (Germany)
**Octocrylene (OCT)**<br>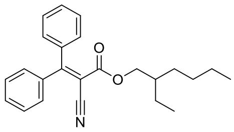<br>Analytical standard, Sigma Aldrich (Germany)	**Avobenzone (AVO)**<br>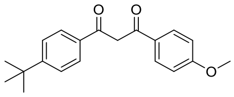<br>Certified reference material, Merck (USA)	**Acesulfame (ACS)**<br>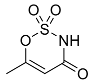<br>*Potassium salt,* ≥ 99%, Fluka (Germany)	**Cyclamate (CAM)**<br>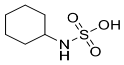<br>*Sodium salt*, p.a. analytical standard, Supelco (USA)
**Neotame (NEO)**<br>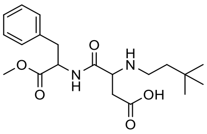<br>Certified reference material, Sigma Aldrich (China)	**2-mercapto-benzothiazole (MBT)**<br>***excluded***<br>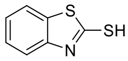<br>> 99.0%, TCI (Japan)	**Caffeine (CAF)**<br>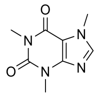<br>≥ 99.0%, Fluka (Switzerland)	**Tris(2-ethylhexyl) phosphate (THP)**<br>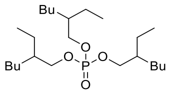<br>94%, Sigma Aldrich (Japan)
**Tris(2-butoxyethyl) phosphate (TBP)**<br>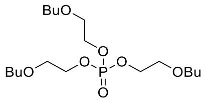<br>97%, Sigma Aldrich (France)	**2-acrylamido-2-methyl-1-propanesulfonic acid (AMA)** ***excluded***<br>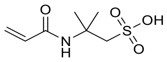<br>97%, Acros Organics (USA)	**Moxifloxacin (MOX)** ****excluded***<br>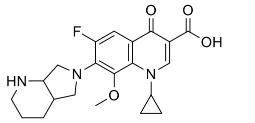<br>*Hydrochloride*, certified reference material, Sigma Aldrich (China)	

SM2, River water characterization and coordinates

Site	Coordinates	pH	Conductivity ($\mu S\ cm^{-1}$)	Overall TIC signal* (ion counts) × 10^9
V1 (spring)[b]	45°50'45.25"N, 13°57'48.38"E	8.2	275	16.0
V2[b]	45°50'41.75"N, 13°56'58.30"E	8.1	312	27.3
V3[b]	45°51'54.45"N, 13°54'30.79"E	7.5	420	37.1
V4[b]	45°52'1.30"N, 13°53'49.67"E	8.3	409	26.5
V5[b]	45°52'23.36"N, 13°49'3.48"E	7.9	383	25.9
V6[b]	45°52'37.54"N, 13°45'13.71"E	8.1	413	40.4
V7[a]	45°53'19.47"N, 13°43'55.35"E	7.5	395	25.1
V8[a]	45°53'35.32"N, 13°42'3.00"E	7.6	391	22.1
V9[a]	45°53'37.56"N, 13°40'14.85"E	7.6	402	17.6
V10[a]	45°53'27.52"N, 13°38'46.61"E	7.9	356	16.7
V11[a]	45°53'24.26"N, 13°37'38.67"E	7.8	424	19.9
V12[a]	45°53'36.83"N, 13°36'49.65"E	8.0	367	18.7
V13[a]	45°53'45.90"N, 13°36'49.78"E	7.6	411	58.0
V14[a]	45°53'28.41"N, 13°35'37.45"E	7.8	359	44.3
Hu-spring[b]	45°54'11.76"N, 13°54'41.83"E	8.1	272	16.2
Li-spring[a]	45°57'30.49"N, 13°43'10.13"E	7.8	261	15.5
Li[a]	45°53'47.98"N, 13°40'53.63"E	7.4	399	29.4
Vr[a]	45°53'54.24"N, 13°37'2.00"E	7.2	388	30.0
Br[b]	45°52'19.26"N, 13°45'54.33"E	8.1	313	25.8

[a]Sample taken on 2 March 2024. [b]Sample taken on 3 March 2024. *Sum of signal intensities per all data points in a TIC LC-MS chromatogram within *m/z* range 200−340 and 350−1200.

SM3, Hydrological data

Location	Temperature (°C)		River flow ($m^3 s^{-1}$)		Water level (cm)	
	2 March	3 March	2 March	3 March	2 March	3 March
V1–V2	9.3	9.3	4.6	5.0	44	57
V6–V7	9.5	9.6	11.1	17.9	96	111
V13–V14	10.5	10.6	14.2	21.3	119	135
Hu*	8.4	8.5	2.8	6.9	60	84
Li	10.5	11.2	0.6	1.8	124	141

Source: Slovenian Environment Agency. *Not monitored at the exact sampling site of the present study - hydrological data was monitored at a middle course of Hubelj river in Ajdovščina city (few kilometres downstream from site Hu-spring).

Average daily values of water levels obtained from the automatic hydrological stations:

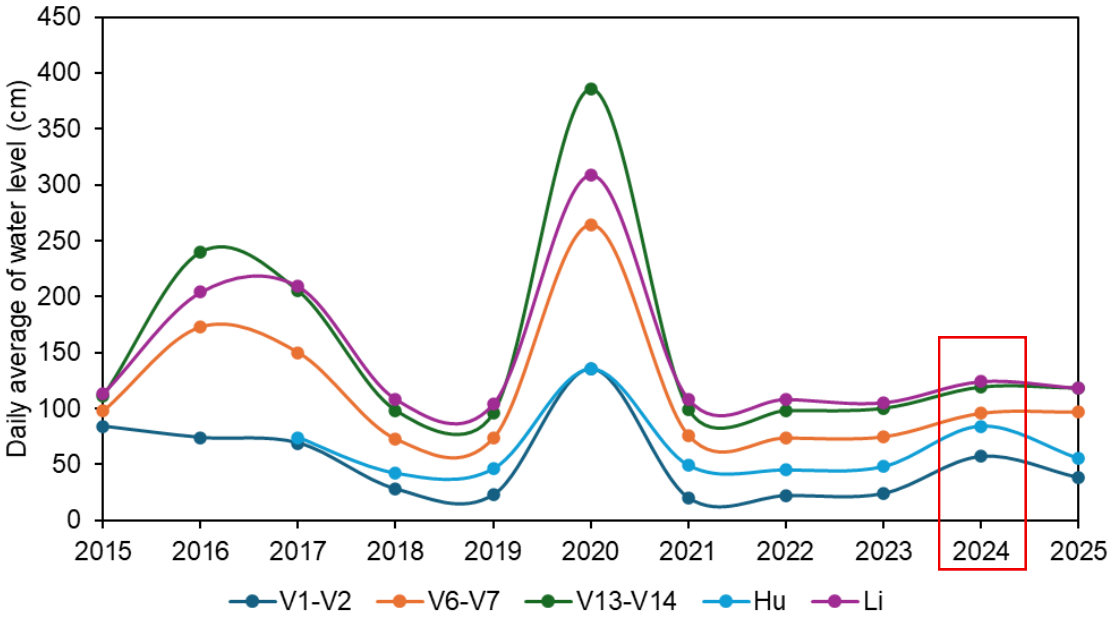

Source: Slovenian Environment Agency, data obtained on May 25, 2025

Comment: The red rectangle marks the value on the day of sampling

SM4, LC-MS/MS methods

Global parameters:

**Chromatographic column**	Phenomenex, Kinetex F5, 100 mm × 3.0 mm, 2.6 µm
**Injection volume**	2 µL
**Flow**	400 µL min^{-1}
**Mobile phases**	A – 0.2% formic acid (Honeywell, LCMS grade) and 1 mM ammonium formate (Sigma-Aldrich, LCMS grade) in MQ water<br>B – 0.2% formic acid and 1 mM ammonium formate in a mixture acetonitrile/methanol (3/2, v/v) (J.T. Baker, LCMS grade / Honeywell, CHROMASOLV)
**Elution programme**	5% B (−1 min), 5% B (0 min), 5% B (3 min), 85% B (13 min), 90% B (18 min), 100% (19−21 min)
**Sheath/Aux/Sweep gas**	5.4/11.3/1.2 L min^{-1} of nitrogen
**ESI probe position**	Centered, medium height, position 1 out of 3 closest to the orifice
**Vaporizer temperature**	350 °C
**Transfer tube temperature**	325 °C

a) Scan parameters for analysis of target CECs:

Global: MRM mode; ESI voltage: −2800/+3800 V; scheduled MRM: time window at retention time ±1.5 min; collision-induced dissociation: 2.0 mtorr of Ar gas; FWHM Q1/Q3: 0.7/0.7 units; dwell time: 50 ms.

Specific:

Analyte	RT* (min)	ESI	Ion type	Precursor (*m/z*)	Products 1 and 2 (*m/z*)**	CE* (V)	RF lens (V)	SF* (V)
AMA	2.4	−	[M−H]$^-$	206	80, 135	19, 28	108, 108	25, 25
ACS	3.5	−	[M−H]$^-$	162	78, 82	32, 12	72, 72	15, 15
CAM	4.1	−	[M−H]$^-$	178	80, 95	25, 32	118, 118	35, 35
CAF	7.5	+	[M+H]$^+$	195	110, 138	23, 19	114, 114	15, 15
PIR	8.3	+	[M+H]$^+$	239	72, 182	20, 16	96, 96	15, 15
ACE	9.5	+	[M+H]$^+$	223	99, 126	37, 21	93, 93	15, 15
RAM	9.6	+	[M+H]$^+$	402	261, 358	24, 20	140, 140	20, 20
MBT	10.7	+	[M+H]$^+$	168	124, 135	26, 22	95, 95	15, 15
NEO	11.2	+	[M+H]$^+$	379	172, 319	23, 18	89, 89	0, 0
RAM	11.2	+	[M+H]$^+$	417	160, 234	30, 21	145, 145	15, 15
TEL	11.9	+	[M+H]$^+$	515	276, 497	47, 35	210, 210	0, 0
ROS	12.0	+	[M+H]$^+$	482	258, 300	32, 35	202, 202	20, 20
CYC	13.9	+	[M+H]$^+$	326	180, 280	21, 13	104, 104	15, 15
TBP	13.9	+	[M+H]$^+$	399	199, 299	16, 13	134, 134	20, 20
SIM	14.3	+	[M+Na]$^+$	441	295, 325	33, 25	162, 162	40, 40
DIF	14.5	+	[M+H]$^+$	395	246, 266	36, 23	141, 141	5, 5
OCT	15.4	+	[M+NH$_4$]$^+$	379	232, 250	23, 13	96, 96	10, 10
AVO	16.3	+	[M+H]$^+$	311	135, 161	22, 22	98, 98	0, 0
THP	17.7	+	[M+CH$_3$CN+Na]$^+$	498	345, 457	23, 11	173, 173	15,15

*RT – retention time, CE – collision energy, SF – source fragmentation. **Quantifier ion is underlined.

b) Scan parameters for analysis of TPs:

Global: MRM mode; ESI voltage: −3800/+3800 V; scheduled MRM: each transition recorded through 2−19 min; collision-induced dissociation: 2.0 mtorr of Ar gas; FWHM Q1/Q3: 0.7/1.2 units; dwell time: 1.5 ms; searched ion type: $[M+H]^+$, except when specified otherwise; collision energy: each transition recorded at 10 and 30 V; RF lens: use calibrated RF lens settings; source fragmentation: 0 V.

Specific:

TP	ESI	Precursor (*m/z*)	Products (*m/z*)	TP	ESI	Precursor (*m/z*)	Products (*m/z*)
PIR87	+	88	56, 71	CYC295	+	296	178, 252, 254, 278
MOX111	+	112	56, 82, 84, 94	CYC297	+	298	210, 228, 254, 280
MBT135	+	136	65, 69, 109,	MBT300	+	301	136.7, 166
PIR153	+	154	83, 98, 137	TEL304	−	303	230, 259, 274, 287
PIR167	+	168	71, 98, 109, 123	CYC313	+	314	250.4, 254, 278, 296
PIR181	+	182	71, 85, 109, 137	MOX320	+	321	233, 275, 303
PIR210	+	211	72, 154, 166	ROS331	+	332	181, 270, 300, 314
CYC211	+	212	107, 130, 133, 194	ROS347a	+	348	241, 258, 270
MBT215	−	214	134, 150	ROS347b	+	348	300, 312, 330
MBT217	+	218	88, 106, 174, 200	ROS349	+	350	201, 242, 256, 270
PIR224	+	225	72, 168, 180, 72	AVO362	+	363	91, 159, 199, 227, 333
MOX251	+	252	174, 202, 216, 234	MOX399	+	400	261, 287, 256, 382
PIR252	+	253	72, 180, 196, 225	ROS403	+	404	282, 300, 368, 386
PIR254	+	255	72, 88, 123, 166, 182	ROS409	+	410	270, 314, 349, 392
PIR238	+	261	188, 223, 243	MOX417	+	418	287, 339, 380, 400
PIR272	+	273	114, 166, 184, 228, 255	ROS425	+	426	286, 299, 330, 364, 408
CYC279	+	280	178, 210, 236, 262	MOX429	+	430	266, 366, 412
CYC281	+	282	155, 182, 238, 254	AVO458	+	459	135, 161, 281, 307, 418
DIF288	+	289	206, 211, 220, 271, 206.6	ROS481	+	482	270, 282, 378, 420, 464
MOX292	+	293	217, 260, 275	TEL512	+	513	209, 276, 289, 303, 483
MOX293	+	294	217, 235, 260, 276	TEL578	+	579	304, 387, 535, 561

More information on TPs is available in: A. Kravos, O. Bajt, H. Prosen, Aquatic photochemistry and transformations of multiclass emerging organic contaminants in environmental water matrices, *Journal of Photochemistry and Photobiology A: Chemistry*, 2025, 469, 116491, DOI: 10.1016/j.jphotochem.2025.116491

c) Scan parameters for chemical fingerprinting

Global: Total-ion current (TIC) scan mode in Q1, ESI voltage: +3800 V; *m/z* range: 200−1200 Da; scan rate: 1000 Da s^{-1}; FWHM: 0.7 units; source fragmentation: 10 V.

SM5, Matrix effects

Analyte	Lower river course[a]					Upper river course[b]				
	$k_{solvent}$	R^2	$k_{extract}$	R^2	ME* (%)	$k_{solvent}$	R^2	$k_{extract}$	R^2	ME* (%)
ACS	350	0.9989	282	0.9892	-19	506	0.9897	400	0.9901	-21
CAM	599	0.9995	493	0.9834	-18	840	0.9974	760	0.9860	-10
CAF	10108	0.9994	10996	0.9844	9	12959	0.9939	16334	0.9949	26
PIR	97470	0.9992	63384	0.9807	-35	138530	0.9957	107102	0.9759	-23
ACE	83768	0.9996	53443	0.9696	-36	119596	0.9953	86591	0.9728	-28
MBT	998	0.9952	610	0.9804	-39	1250	0.9914	226	0.9574	-82
MOX	2640	0.9804	6910	0.9788	162	2035	0.9791	999	0.9873	-51
RAM	137801	0.9991	121074	0.9710	-12	198147	0.9940	197025	0.9760	-1
NEO	15577	0.9986	41691	0.9660	168	22657	0.9947	63501	0.9760	180
CYC	26982	0.9996	15352	0.9579	-43	38008	0.9931	21484	0.9794	-43
TEL	78531	0.9985	52693	0.9652	-33	111996	0.9950	95630	0.9902	-15
ROS	5993	0.9982	4470	0.9630	-25	8852	0.9926	7702	0.9867	-13
TBP	120293	0.9993	91326	0.9660	-24	169877	0.9958	150430	0.9805	-11
SIM	18749	0.9998	11221	0.9652	-40	24184	0.9953	18654	0.9822	-23
DIF	6634	0.9995	3544	0.9562	-47	9175	0.9960	6050	0.9745	-34
OCT	8127	0.9992	4155	0.9557	-49	10250	0.9805	1125	0.9903	-89
AVO	45593	0.9988	21087	0.9834	-54	58153	0.9877	47369	0.9694	-19
THP	29302	0.9994	15899	0.9826	-46	38116	0.9966	29841	0.9879	-22

*ME = [$k_{solvent}/k_{extract}$] × 100. [a]Lower river course applies to locations: V7–V14, Li, Vr. [b]Upper river course applies to locations: V1–V6, Br, Hu-spring, Li-spring.

SM6, LC-QTOF method

**Chromatographic column**	Waters, Acquity Premier BEH C18, 50 mm × 2.1 mm, 1.7 µm
**Injection volume**	2 µL
**Flow**	500 µL min^{-1}
**Mobile phases**	A – 0.1% formic acid (Honeywell, LCMS grade) in MQ water<br>B – 0.1% formic acid in acetonitrile (J.T. Baker, LCMS grade)
**Elution programme**	10% B (0 min), 30% B (5 min), 90% B (8 min), 90% B (9 min), 10% B (9.5–10 min)
**Capillary voltage**	+1.0 kV
**Sampling cone**	40 V
**Source temperature**	120 °C
**Desolvation temp.**	550 °C
**Desolvation gas flow**	1000 L h^{-1}
**Cone gas flow**	50 L h^{-1}
**LockMass correction**	LockSpray acquired by analysing 100 pg μL^{-1} Leucine enkephalin ($C_{28}H_{37}N_5O_7$, *m/z* 556.2771) standard solution (Waters)
**QTOF calibration**	Low-mass calibration (100−1000 *m/z*) was done prior every analysis via infusion of sodium formate standard solution (Waters)
**MS^E continuum scan mode (also known as all-ion fragmentation or data-independent acquisition)**	Positive polarity, sensitivity analyser mode, normal dynamic range, MS^E acquired over the range 100−1000 Da, 0.1 s scan time, continuum data format, collision energy switching between 0 V (for low-energy TICs) and 20−30 V ramping (for high-energy TICs)

SM7, Panoramic view over Vipava valley from its northern edge

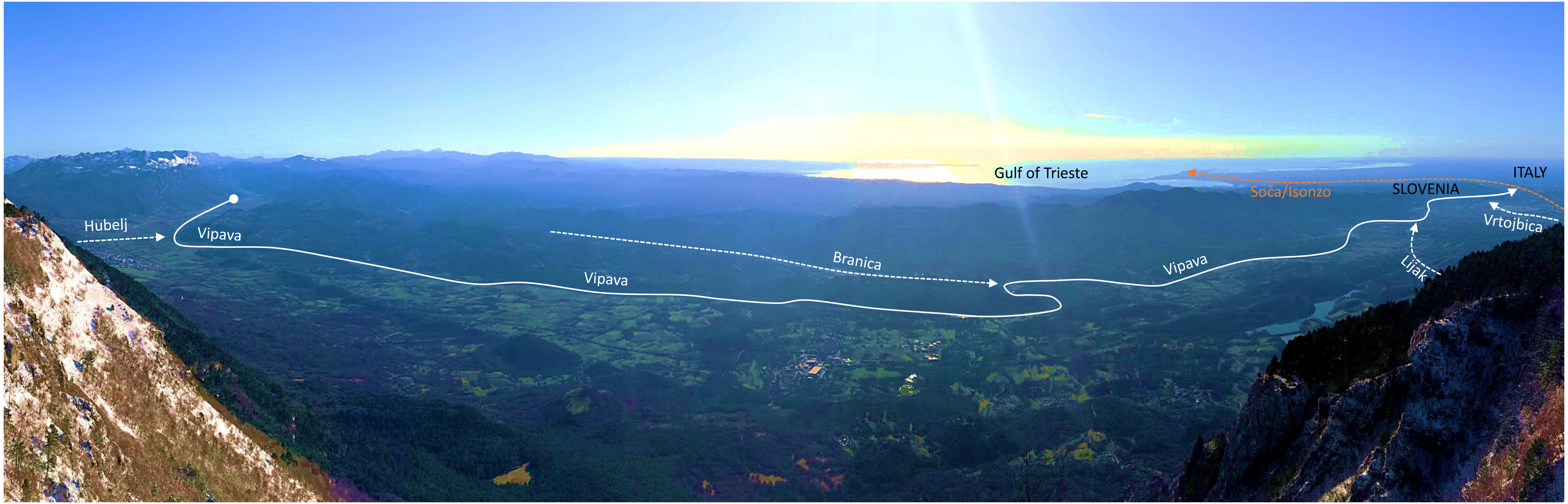

Photo taken by Aleksander Kravos – view from a mountain peak Veliki Rob, Trnovski gozd, December 2024. DISCLAIMER: The riverways are sketched and not drawn in detail

SM8, Determined concentrations and risk quotient (RQ) labelling

River	Site	Concentration in river water (ng L^{-1}) and RQ labelling* (colours)														
		ACS	CAM	CAF	PIR	ACE	RAM	NEO	TEL	ROS	TBP	SIM	DIF	THP	OCT**	AVO**
Vipava	V1	<LOD	<LOD	6.2	<LOD	<LOD	<LOD	<LOD	<LOD	<LOD	<LOD	<LOD	<LOD	<LOD	<LOD	<LOD
Vipava	V2	<LOD	<LOD	14.8	<LOD	<LOD	<LOD	<LOD	<LOQ	<LOQ	<LOD	<LOD	<LOD	<LOD	<LOD	<LOD
Vipava	V3	67.0	39.4	175.1	<LOQ	<LOD	<LOD	<LOD	4.6	<LOQ	20.3	<LOD	<LOD	565.0	69.9	64.4
Vipava	V4	150.4	57.5	105.3	<LOQ	<LOD	<LOD	<LOD	25.3	16.7	7.4	<LOD	67.3	363.4	<LOD	57.9
Vipava	V5	<LOD	<LOD	89.6	<LOQ	<LOD	<LOD	<LOD	6.6	1.0	5.2	<LOD	<LOD	44.3	<LOQ	19.1
Vipava	V6	27.6	4.8	14.8	<LOQ	<LOD	<LOD	<LOD	<LOQ	<LOQ	1.4	<LOD	15.7	108.4	<LOD	10.2
Vipava	V7	21.3	6.6	36.5	<LOQ	<LOD	<LOD	<LOD	<LOQ	<LOQ	2.8	<LOD	<LOD	199.7	<LOD	9.6
Vipava	V8	6.7	4.7	53.1	<LOD	<LOD	<LOD	<LOD	39.1	<LOQ	8.9	<LOD	12.6	<LOD	<LOD	<LOQ
Vipava	V9	5.3	<LOD	56.9	<LOD	<LOD	<LOD	<LOD	13.3	2.8	3.0	<LOD	<LOD	<LOD	<LOD	<LOQ
Vipava	V10	5.1	<LOD	43.9	<LOQ	<LOD	<LOD	<LOD	9.1	<LOQ	3.5	<LOD	<LOD	<LOQ	<LOD	6.2
Vipava	V11	49.1	15.1	140.9	<LOQ	<LOD	<LOD	<LOD	9.4	1.9	4.7	<LOD	<LOD	<LOD	<LOD	4.5
Vipava	V12	3.5	<LOD	37.2	<LOQ	<LOD	<LOD	<LOD	11.2	1.5	3.4	<LOD	<LOD	<LOD	<LOD	<LOQ
Vipava	V13	5.0	<LOD	82.8	<LOQ	<LOD	<LOD	<LOD	47.5	1.9	9.7	<LOD	<LOD	67.4	<LOQ	13.3
Vipava	V14	29.1	18.1	307.0	<LOD	<LOD	<LOD	<LOD	72.9	18.3	7.8	<LOD	<LOD	<LOD	<LOD	2.4
Lijak	Li_{spring}	<LOD	<LOD	8.8	<LOD	<LOD	<LOD	<LOD	<LOD	<LOD	<LOD	<LOD	<LOD	<LOD	<LOD	<LOD
Lijak	Li	26.9	17.9	118.4	<LOQ	<LOD	<LOD	<LOD	<LOQ	8.2	9.8	<LOD	30.2	<LOD	<LOD	<LOD
Hubelj	Hu_{spring}	<LOD	<LOD	<LOQ	<LOD	<LOD	<LOD	<LOD	<LOD	<LOD	<LOD	<LOD	<LOD	<LOD	<LOD	<LOD
Branica	Br	2.8	<LOD	42.7	<LOQ	<LOD	<LOD	<LOD	7.7	<LOD	2.0	<LOD	<LOD	<LOQ	<LOD	8.9
Vrtojbica	Vr	76.0	31.8	127.6	<LOQ	<LOD	<LOD	<LOD	11.3	1.6	19.8	<LOD	<LOD	434.6	54.1	63.4
LOD (ng L^{-1}) =		0.8	3.5	2.0	0.1	4.4	1.0	0.5	0.8	0.3	0.8	4.7	0.8	5.9	13.9	0.8
LOQ (ng L^{-1}) =		2.4	11.0	6.0	0.3	13.1	2.9	1.6	2.3	0.8	2.9	14.1	2.3	17.6	41.6	2.3
PNEC (ng L^{-1}) =		72,400	18,500	1,200	90	37	1×10^6	1,370	49,000	1,800	24,000	2,630	10	39	270	120

*Colouration and RQ labelling: RQ < 1, RQ 1–5, RQ 5–10, RQ > 10; RQ = c/PNEC (c … concentration in river water, PNEC … lowest predicted no-effect concentration)
**Qualitative analysis - concentration in a river water extract (µg L^{-1}).

SM9, TP identification with LC-MS/MS

a) Alignment of retention times in pseudo-reference vs. river extract

Shown extracted ion chromatograms (EICs) for the most abundant MRM transition

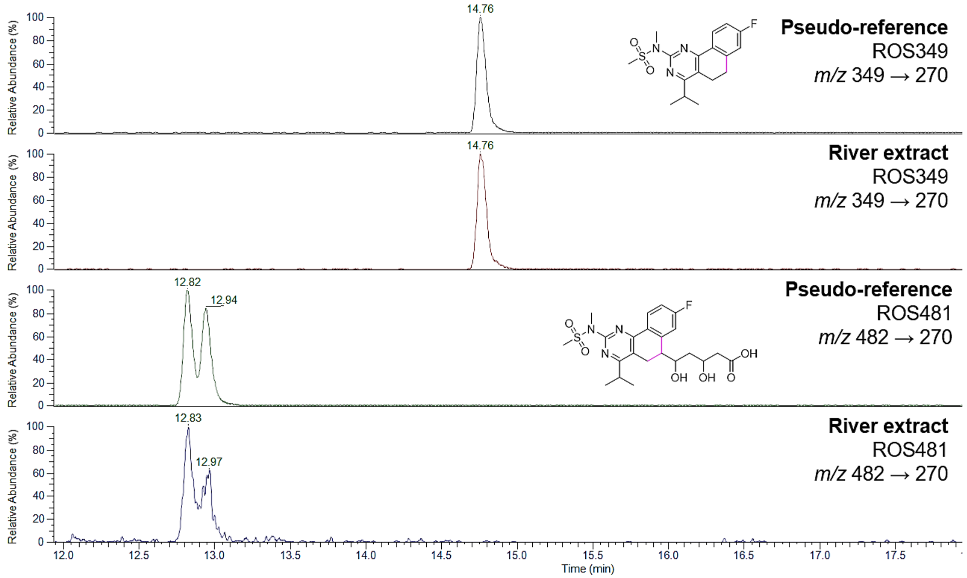

b) EICs for all detected MRM transitions

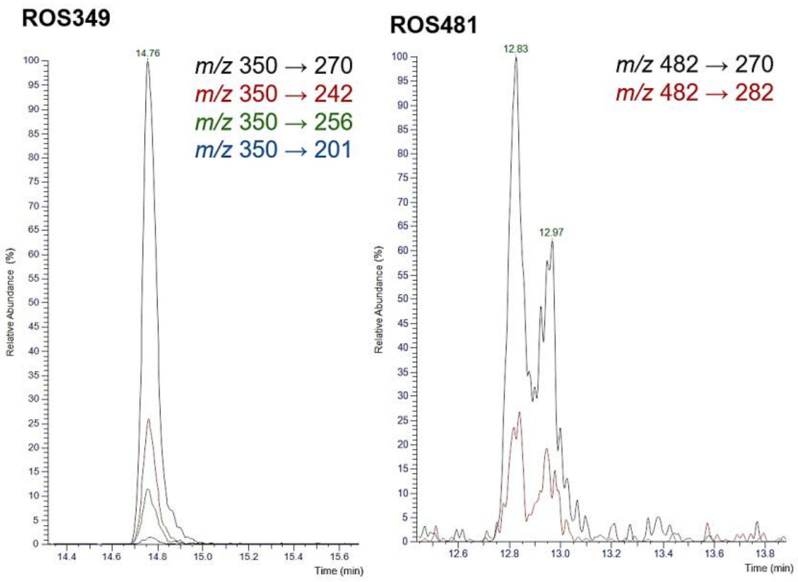

c) Ion ratios* for detected MRM transitions

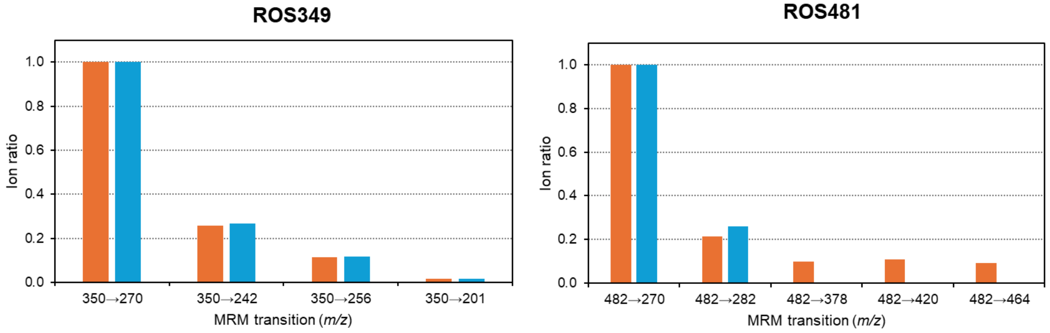

Legend: Pseudo-reference River water extract

*Ion ratio = [signal intensity for a given MRM transition]/[signal intensity for the most abundant (first) MRM transition]

SM10, TP identification with LC-QTOF

QTOF MS/MS spectrum for ROS349

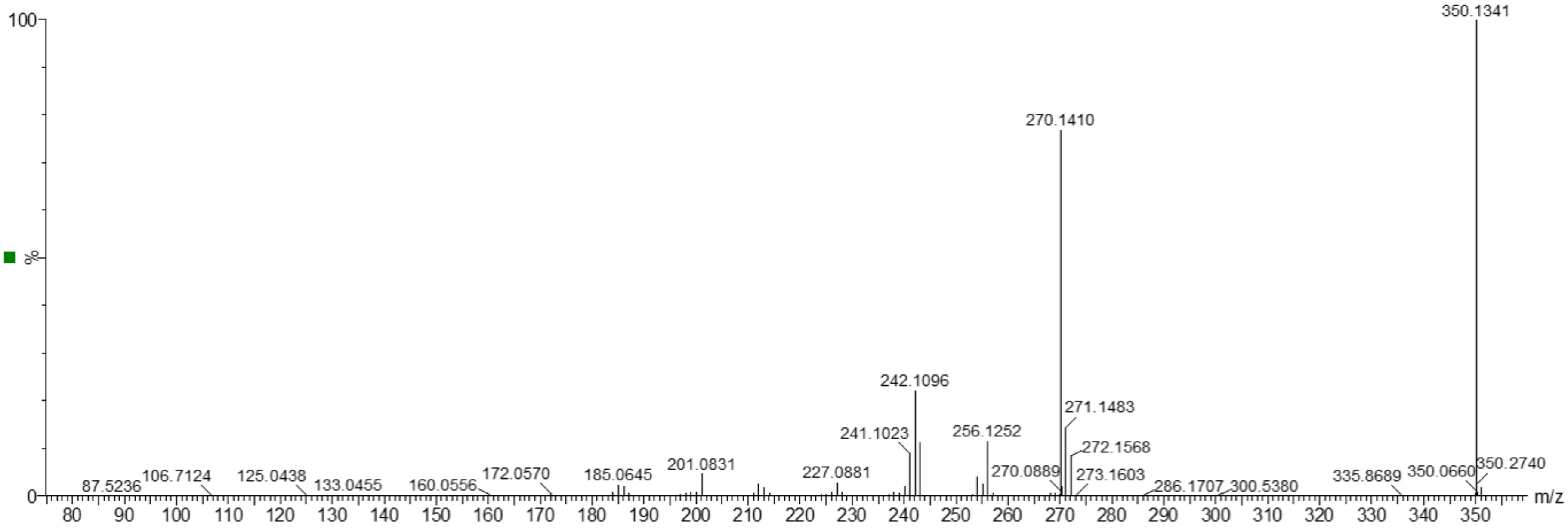

Observed *m/z* of precursor (molecular ion) for ROS349 in pseudo-reference (top spectrum, *m/z* = 350.1345, *Δ* = 3.42 ppm) and in river water extract (bottom spectrum, *m/z* = 350.1336, *Δ* = 0.85 ppm)

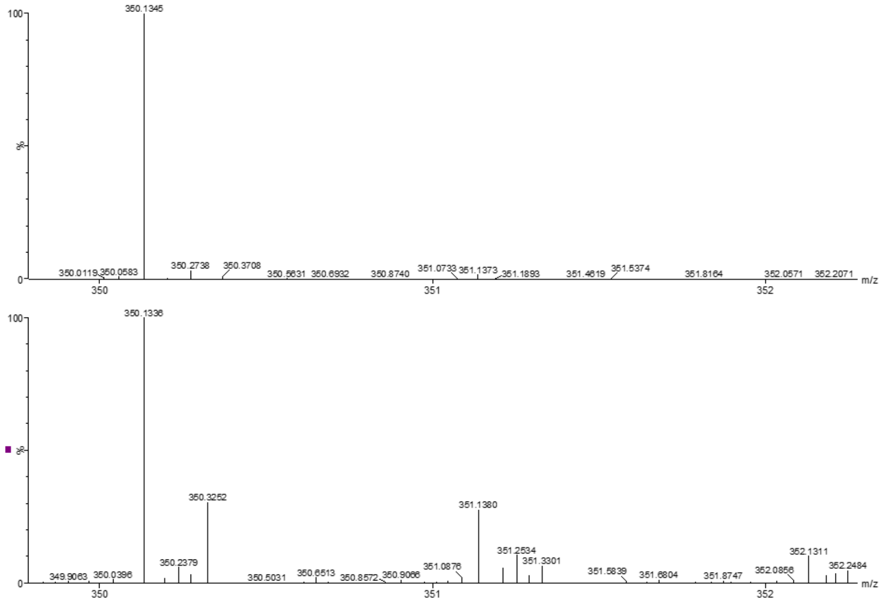

Observed *m/z* of fragment 270 *m/z* ($C_{16}H_{17}N_3F^+$) in pseudo-reference (top spectrum, *m/z* = 270.1410, *Δ* = 3.32 ppm) and in river water extract (bottom spectrum, *m/z* = 270.1402, *Δ* = 0.36 ppm)

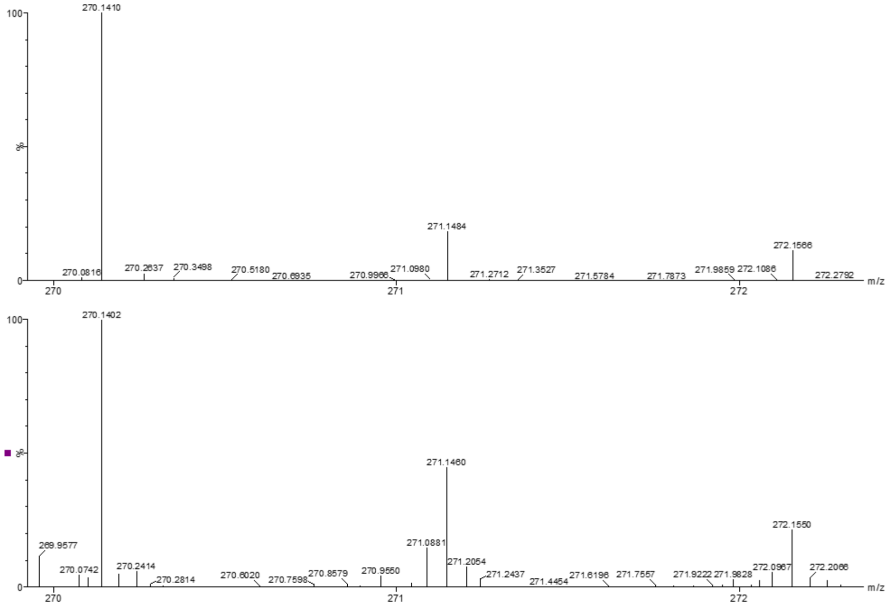

**Observed *m/z* of fragment 242 *m/z* ($C_{14}H_{13}N_3F^+$) in pseudo-reference (top spectrum, *m/z* = 242.1099, *Δ* = 4.53 ppm) and in river water extract (bottom spectrum, *m/z* = 242.1093, *Δ* = 2.06 ppm)**	**Observed *m/z* of fragment 256 *m/z* ($C_{15}H_{15}N_3F^+$) in pseudo-reference (top spectrum, *m/z* = 256.1254, *Δ* = 3.70 ppm) and in river water extract (bottom spectrum, *m/z* = 256.1250, *Δ* = 2.14 ppm)**
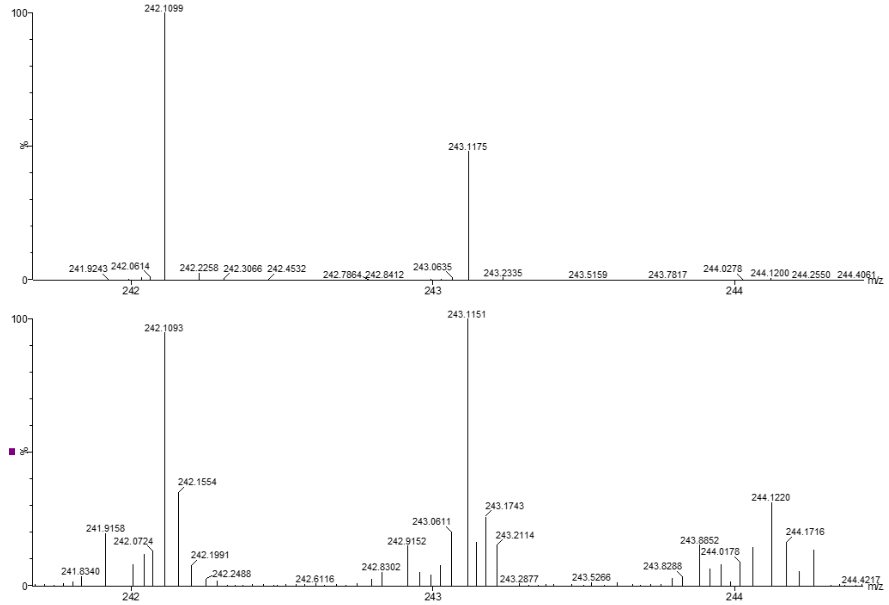	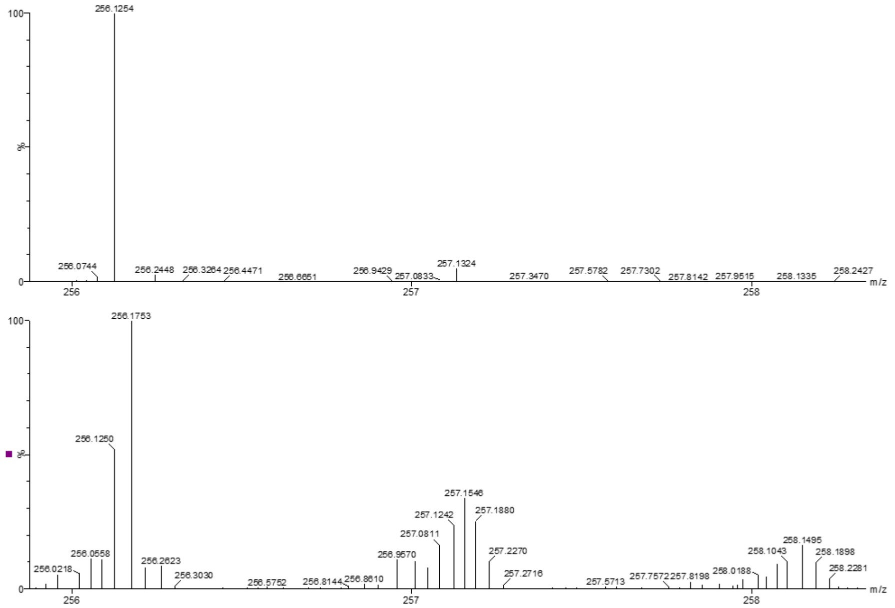

QTOF MS/MS spectrum for ROS481

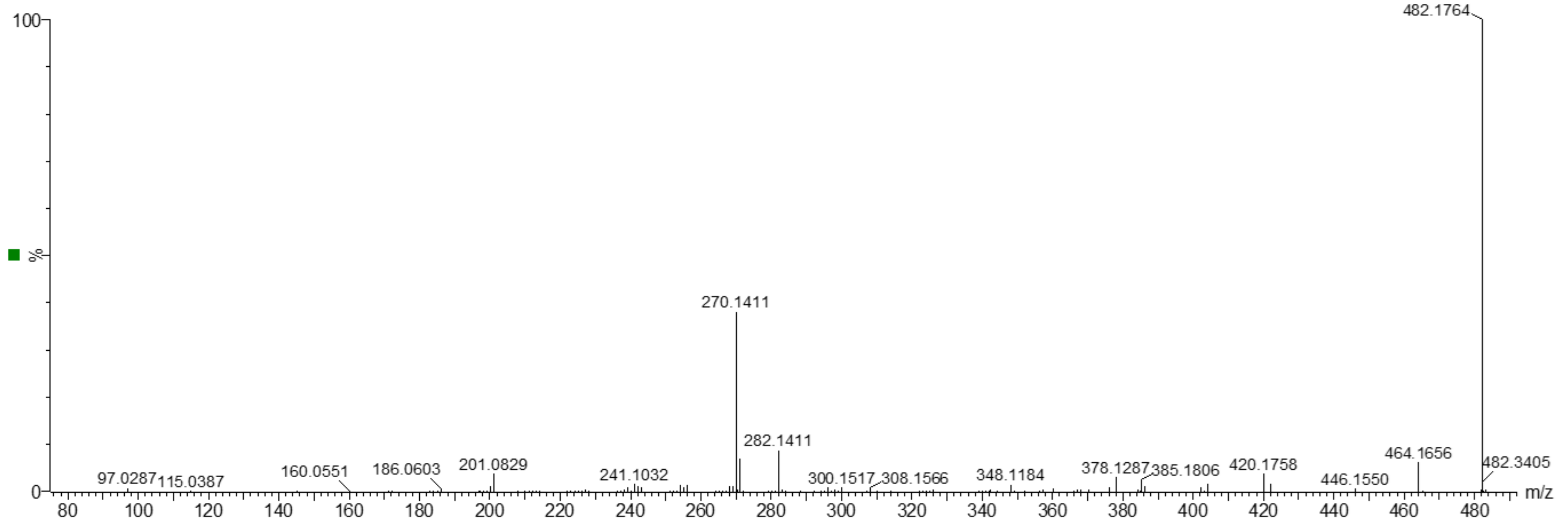

Observed *m/z* of precursor (molecular ion) for ROS481 in pseudo-reference (top spectrum, *m/z* = 482.1764, *Δ* = 1.74 ppm) and in river water extract (bottom spectrum, *m/z* = 482.1761, *Δ* = 1.18 ppm)

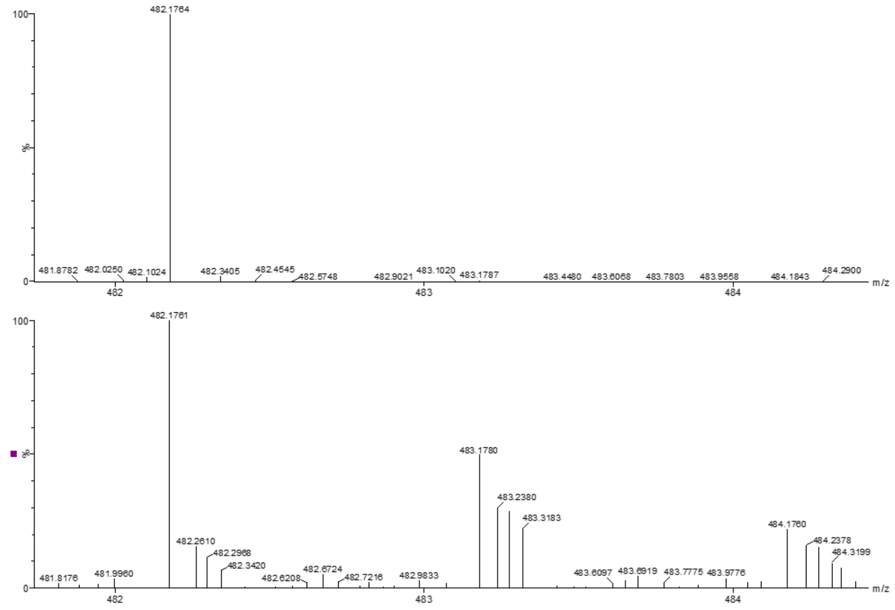

Supplement: Supplementary file 1 — Supplementary Material 1 (PDF 1.16 MB) [file 10661_2026_15437_MOESM1_ESM.pdf]
